# The *Elovl4* Spinocerebellar Ataxia-34 Mutation 736T>G (p.W246G) Impairs Retinal Function in the Absence of Photoreceptor Degeneration

**DOI:** 10.1007/s12035-020-02052-8

**Published:** 2020-08-11

**Authors:** Martin-Paul Agbaga, Megan A. Stiles, Richard S. Brush, Michael T. Sullivan, Adeline Machalinski, Kenneth L. Jones, Robert E. Anderson, David M. Sherry

**Affiliations:** 1 Dean McGee Eye Institute, University of Oklahoma Health Sciences Center, Oklahoma City, OK 73104 USA; 2 Department of Ophthalmology, University of Oklahoma Health Sciences Center, 608 Stanton L. Young Blvd, DMEI 428PP, Oklahoma City, OK 73104 USA; 3 Department of Cell Biology, University of Oklahoma Health Sciences Center, 940 SL Young Blvd, BMSB-536, Oklahoma City, OK 73104 USA; 4 Oklahoma Center for Neurosciences, University of Oklahoma Health Sciences Center, Oklahoma City, OK 73104 USA; 5 Harold Hamm Diabetes Center, University of Oklahoma Health Sciences Center, Oklahoma City, OK 73104 USA; 6 Department of Pharmaceutical Sciences, University of Oklahoma Health Sciences Center, Oklahoma City, OK 73104 USA

**Keywords:** Very long chain fatty acids, Neurodegeneration, Stargardt-like macular dystrophy, Retina, Cerebellum

## Abstract

*Elo*ngation of *v*ery *l*ong chain fatty acids-*4* (ELOVL4) is essential for synthesis of very long chain polyunsaturated and saturated fatty acids (VLC-PUFA and VLC-SFA, respectively) of chain length greater than 26 carbons. Mutations in the *ELOVL4* gene cause several distinct neurodegenerative diseases including Stargardt-like macular dystrophy (STGD3), spinocerebellar ataxia 34 (SCA34), and a neuro-ichthyotic syndrome with severe seizures and spasticity, as well as erythrokeratitis variabilis (EKV), a skin disorder. However, the relationship between *ELOVL4* mutations, its VLC-PUFA and VLC-SFA products, and specific neurological symptoms remains unclear. We generated a knock-in rat line (SCA34-KI) that expresses the 736T>G (p.W246G) form of *ELOVL4* that causes human SCA34. Lipids were analyzed by gas chromatography and mass spectrometry. Retinal function was assessed using electroretinography. Retinal integrity was assessed by histology, optical coherence tomography, and immunolabeling. Analysis of retina and skin lipids showed that the W246G mutation selectively impaired synthesis of VLC-SFA, but not VLC-PUFA. Homozygous SCA34-KI rats showed reduced ERG a- and b-wave amplitudes by 90 days of age, particularly for scotopic responses. Anatomical analyses revealed no indication of neurodegeneration in heterozygote or homozygote SCA34-KI rats out to 6–7 months of age. These studies reveal a previously unrecognized role for VLC-SFA in regulating retinal function, particularly transmission from photoreceptors to the inner retina, in the absence of neurodegeneration. Furthermore, these findings suggest that the tissue specificity and symptoms associated with disease-causing *ELOVL4* mutations likely arise from selective differences in the ability of the mutant ELOVL4 enzymes to support synthesis of VLC-PUFA and/or VLC-SFA.

**Electronic supplementary material:**

The online version of this article (10.1007/s12035-020-02052-8) contains supplementary material, which is available to authorized users.

## Introduction

*Elo*ngation of *v*ery *l*ong chain fatty acids-*4* (ELOVL4) catalyzes the first, rate-limiting step in the synthesis of very long chain polyunsaturated and saturated fatty acids with fatty acid chain lengths of 28 carbons or more [VLC-PUFA and VLC-SFA, respectively [1–3]]. ELOVL4 is the only known fatty acid elongase that performs this function [1, 2]. Expression of ELOVL4 is limited to a small number of organs including the retina, brain, skin, testes, and Meibomian gland [4–6], which show tissue-specific VLC-PUFA and VLC-SFA profiles. Our meticulous recent lipidomic analysis of mouse [7], rat, and baboon [3] brains using modern analytical tools found VLC-SFA (28:0 and 30:0). These VLC-SFA were incorporated into complex sphingolipids and were enriched in synaptic vesicles prepared from baboon hippocampus, where they contribute to the regulation of presynaptic neurotransmitter release [3, 8]. Previous studies have reported phosphatidylcholine containing VLC-PUFA in early postnatal rat brain and in the brains of human patients with Zellweger’s disease, a peroxisomal disorder that disrupts lipid metabolism [9, 10], but no VLC-PUFA have been reported in the healthy mature brain. VLC-SFA are also found in the Meibomian glands and skin, where they are incorporated into ω-O-acylceramides and form the water barrier of the tear film and skin [5, 11–14]. VLC-PUFA are present in the outer segments of rod and cone photoreceptors in the retina esterified to the *sn-1* position of phosphatidylcholine [15–18]. VLC-PUFA in testes [19–21] are present in an amide linkage to sphingolipids [22–24].

ELOVL4 products have emerged as important novel regulators of synaptic function and neuronal survival in the CNS [23]. Several different mutations in the *ELOVL4* gene cause neurological diseases that vary according to the mutation and its inheritance pattern [22, 25–36]. Heterozygous inheritance of four different *ELOVL4* mutations causes autosomal dominant spinocerebellar ataxia-34 (SCA34), a late-onset neurodegenerative disease of the cerebellum that results in a characteristic gait ataxia that may be accompanied by dysarthria and eye movement abnormalities, with or without erythrokeratitis variabilis (EKV), a disorder of the skin [28–30, 35]. Patients with SCA34 have no reported clinical retinal phenotype [28–30, 35]. However, a recent report identified an American family with a novel *ELOVL4* mutation (c.512T>C,p.I171T) that causes spinocerebellar ataxia with some family members also showing retinitis pigmentosa [37].

Heterozygous inheritance of six different *ELOVL4* mutations causes autosomal dominant Stargardt-like macular dystrophy (STGD3), which typically presents with juvenile onset [26, 27, 31–33, 36]. Patients with STGD3 have no other reported central nervous system or skin phenotypes [26, 27, 31–33, 36]. Homozygous inheritance of three other recessive *ELOVL4* mutations causes a devastating neuro-ichthyotic syndrome characterized by seizures, intellectual disability, spasticity, ichthyosis, and premature death [25, 34]. Homozygous inheritance of the five-base-pair STGD3 mutation in mice is neonatal lethal due to dehydration caused by the absence of ω-O-acyl-cerebrosides, a product of ELOVL4 that provides the permeability barrier [11–14]. Conditional expression of ELOVL4 in skin rescued the neonatal lethality, but the mice developed a severe seizure syndrome at postnatal day 18 similar in many respects to the recessive human ELOVL4 neuro-ichthyotic syndrome [3]. No human patients with homozygous inheritance of STGD3 or SCA34 alleles have been reported.

Spinocerebellar ataxias comprise a large constellation of autosomal dominant hereditary disorders that are characterized by progressive discoordination of gait (ataxia) and cerebellar atrophy. Spinocerebellar ataxias arise from defects in a large number of different genes, which produce distinct types of ataxia with characteristic times of onset and rates of disease progression [38, 39]. In addition to the characteristic ataxia, SCA patients also may show additional symptoms including dysarthria (difficulty speaking), apraxia (loss of fine motor control), eye movement deficits, and abnormal spinal reflexes, depending on the specific form of the disease. Currently, there are no treatments for any form of the disease.

How the different mutations in *ELOVL4* affect levels of VLC-SFA and VLC-PUFA that cause distinct tissue-specific pathologies remains unclear. A key gap in our understanding is whether these disease-causing *ELOVL4* mutations also compromise the function of additional tissues. To better understand the role of ELOVL4 and its VLC-fatty acid products in the retina, we generated a novel knock-in rat model expressing the 736T>G (p.W246G) form of *ELOVL4* that causes human SCA34 [35] and examined lipid composition, retinal function, and photoreceptor degeneration. Our studies reveal a previously unrecognized role for the VLC-fatty acid products of ELOVL4 in synaptic transmission from photoreceptors to the inner retina in the absence of photoreceptor degeneration. This deficit is most likely attributable to deficiency of VLC-SFA rather than VLC-PUFA.

## Methods

### Animals

#### Generation and Characterization of c.736T>G, p.W246G Knock-In Rats Using CRISPR/Cas9

**Fig. 1:**
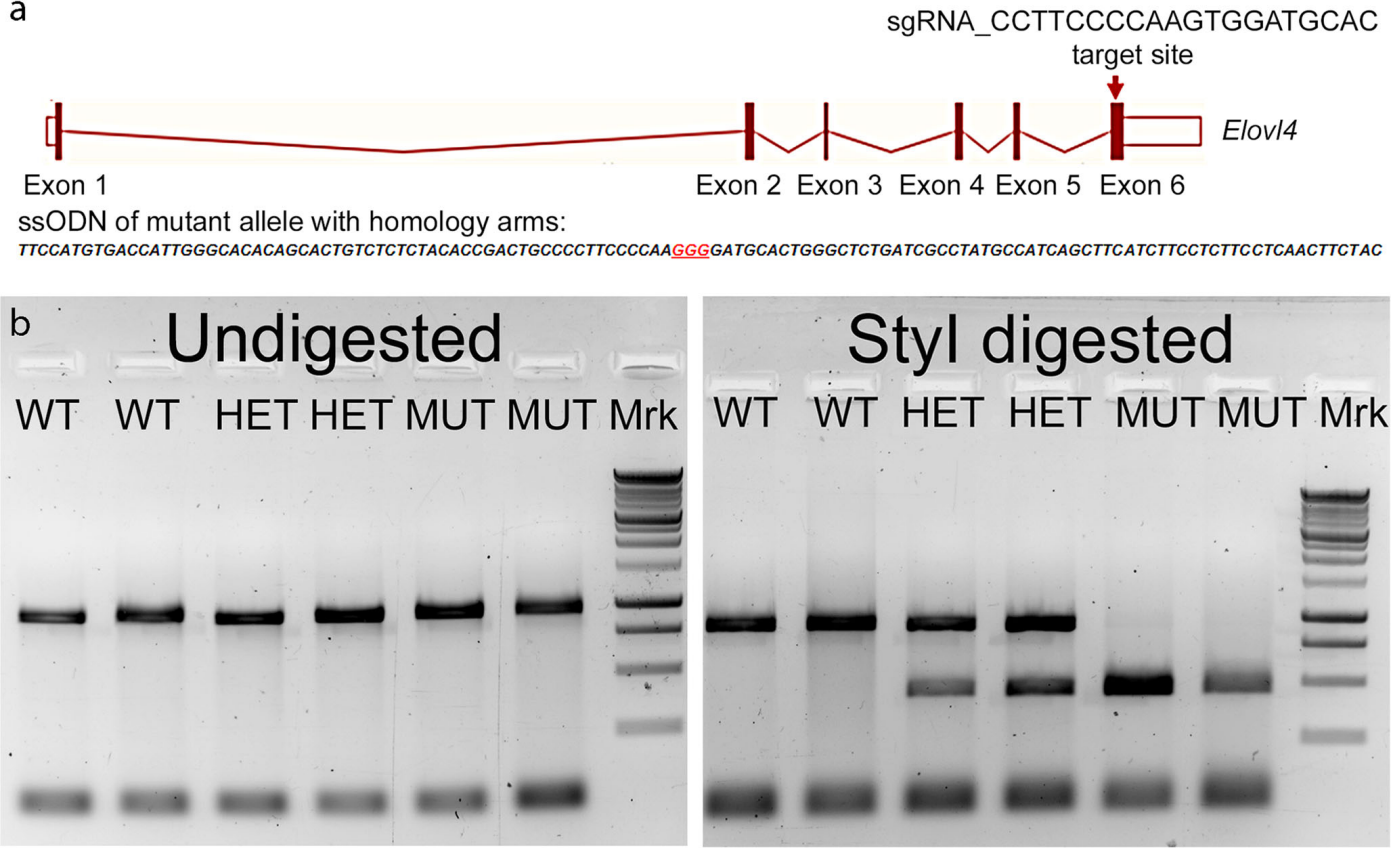
Generation and characterization of c.736T>G, p.W246G knock-in Long-Evans rat using the CRISPR/Cas9 system. **a** Schematic depiction of targeting strategy. The genomic region of the rat *Elovl4* locus is diagrammed (the gene is oriented from left to right; total size is 28.21 kb). Solid bars represent open reading frame (exons). Open bars represent untranslated regions. The sgRNA cut site and the single-strand oligonucleotide donor sequencing with homology arms and the mutation site is shown by the red arrow. **b** Genotyping of WT, HET, and MUT SCA34-KI rats by StyI restriction analysis. Seven hundred four base–pair PCR products were generated from WT, HET, and MUT rats using PCR primers. The amplicons were run undigested or purified and digested with StyI restriction enzyme. Left panel: undigested amplicons ran at 704 bp for WT, HET, and MUT rats, as expected. Right panel: digestion with StyI restriction enzyme showed the WT PCR product is resistant to Sty I digestion. In contrast, HET rats show two bands, one corresponding to the WT PCR product and a smaller, digested fragment arising from the mutant PCR product containing the StyI digestion site. MUT rats show only a single band corresponding to the digested, mutant PCR product with no WT PCR product, as expected

The CRISPR/Cas9 genetic editing approach was used to generate a heterozygous Long-Evans rat model of human SCA34 described by Ozaki et al. [35]. To minimize potential off-target effects, human Cas9 D10A (hCas9-D10A) nickase was used to knock-in the c.736T>G mutation by targeting exon 6 of the rat *Elovl4* (Ensembl: ENSRNOG00000009773) located on rat chromosome 8 (Fig. 1a). The hCas9 mRNA, sgRNA (CCTTCCCCAAGTGGATGCAC), and single-strand oligonucleotide donor (ssODN: *TTCCATGTGACCATTGGGCACACAGCACTGTCTCTCTACACCGACTGCCCCTTCCCCAAG****GGG****ATGCACTGGGCTCTGATCGCCTATGCCATCAGCTTCATCTTCCTCTTCCTCAACTTCTAC)* were co-injected into Long-Evans rat zygotes at the laboratories of Cyagen Bioscience Inc. (Santa Clara, CA). From the injected embryos transplanted into foster mothers, three heterozygous F0 rats carried the c.736T>G, p.W246G mutant SCA34 gene. The rats were genotyped using *rat Elovl4-F: 5′-GCCCTTCAGCGAGTATTCAAATGTC-3′* and *Elovl4-R: 5′-GGTCACGTTGGGATT GCTTTGGTCTGGA-3′* PCR primers that generate a 900-base-pair (bp) product, which upon restriction digestion with StyI (New England Biolabs, Ipswich, MA), generates a 450-base-pair product that allows identification of heterozygous and homozygous rats (Fig. 1b) due to introduction of the StyI restriction site from the c.736T>G mutation. The wild-type (WT) 900-base-pair band is resistant to the Sty I digestion (Fig. 1b). We bred the F0 rats with WT Long-Evans rats over several generations to breed out any potential off-target effects and to establish the heterozygous SCA34 Long-Evans rat model. We also successfully generated homozygous SCA34 rats (MUT) by breeding heterozygous rats from the different F0 lines. Knock-in of the SCA34 mutation in the rats was further verified by Sanger sequencing at Oklahoma Medical Research Foundation, Oklahoma City, OK) (Supplemental Fig. 1) and by whole-genome DNA sequencing (Novogene Corporation, Inc., Sacramento, CA) (Supplemental Fig.2).

Subsequent studies were performed using WT, heterozygous (HET), and homozygous (MUT) SCA34-knock-in Long-Evans rats (SCA34-KI) carrying the c.736T>G (p.W246G) mutation in *ELOVL4* that causes age-related SCA34 in humans [35]. The rats were maintained in a pathogen-free barrier facility on a 12-h light:12-h dark daily light cycle (~ 25–40 lx at cage level). Food and water were available at all times. All animal procedures were approved by the University of Oklahoma Health Sciences Center Institutional Animal Care and Use Committee and conformed to the National Institutes of Health Guide for the Care and Use of Laboratory Animals, the Association for Research in Vision and Ophthalmology Resolution on the Use of Animals in Research, and US Public Health Service guidelines.

#### Lipid Analysis

Skin VLC-SFA were quantified using gas chromatography-mass spectrometry (GC-MS) as previously described [1]. Briefly, 50 mg of tissue was homogenized in methanol and the lipids were extracted [40] and converted to fatty acid methyl esters (FAME) as described previously [41]. The GC-MS was operated in the electron impact (EI) single-ion monitoring (SIM) mode. The 26:0, 28:0, and 30:0 response values were obtained using the *m*/*z* ratios 74.1 and 87.1 as well as 410.4, 438.4, and 466.5, respectively. Sample concentrations were determined by comparison to external standards, using 25:0 and 27:0 as internal standards.

Retinal phospholipids were measured using electrospray ionization-mass spectrometry-mass spectrometry (ESI-MS-MS) as previously described [8, 42]. Briefly, tissue was homogenized in 40% aqueous methanol and diluted 1:40 with 2-propanol/methanol/chloroform (4:2:1) containing 20 mM ammonium formate and 1.0 μM phosphatidylcholine (PC, 14:0/14:0), 1.0 μM phosphatidylethanolamine (PE, 14:0/14:0), and 0.33 μM phosphatidylserine (PS, 14:0/14:0) as internal standards. Samples were introduced into a TSQ Ultra mass spectrometer using an Advion NanoMate operating in infusion mode. PC lipids were measured using precursor ion scanning of *m*/*z* 184, PE lipids (including plasmalogens) were measured using neutral loss scanning of *m*/*z* 141, and PS lipids were measured using neutral loss scanning of *m*/*z* 185. All species detected for each group were represented as a relative percentage of the sum based on their response values.

#### Electroretinography

Flash ERGs were recorded using a Diagnosys Espion E2 ERG system (Diagnosys LLC, Lowell, MA). The rats were maintained overnight in total darkness and prepared for ERG recording under dim red light. The rats were anesthetized with an intraperitoneal injection of ketamine (100 mg/kg body weight) and xylazine (5 mg/kg body weight). A drop of 0.5% (v/v) proparacaine HCl was applied to the cornea for local anesthesia. A drop of 10% (v/v) phenylephrine was applied to the cornea to dilate the pupil. The rats were kept on a heating pad at 37 °C during recording. A gold recording electrode was placed on the cornea, with a reference electrode positioned in the mouth and a ground electrode in the tail, and the rats were placed inside a Ganzfeld illuminating sphere. Responses were differentially amplified, averaged, and stored. To assess rod photoreceptor function (scotopic ERG), five strobe flash stimuli were presented at flash intensities of 0.004, 5.635, 11.243, 22.433, and 178.92 cd·s/m^2^. Scotopic a-wave amplitude was measured from the pre-stimulus base line to the negative trough of the a-wave. The scotopic b-wave amplitude was measured from the trough of the a-wave to the positive peak of the b-wave. To evaluate cone function (photopic ERG), a strobe flash stimulus (3.3 log cd s/m^2^) was presented to dilated, light-adapted (5 min at 1.7 log cd s/m^2^) rats. Photopic, short-wavelength cone, middle-wavelength cone, and flicker (3, 10, 20, and 30) Hz ERG responses were recorded, and the amplitude of the cone b-wave was measured from the trough of the a-wave to the peak of the b-wave.

#### Optical Coherence Tomography

Optical coherence tomography (OCT) was used to acquire images of retinal layering in vivo. OCT analysis was performed using a Bioptigen SDOIS system (Leica Microsystems, Buffalo Grove, IL). Rats were anesthetized by intraperitoneal injection of ketamine (100 mg/kg body weight) and xylazine (5 mg/kg body weight), and a drop of 10% (v/v) phenylephrine was applied to the cornea to dilate the pupil. The animal was placed in the animal mount, the eyes were well hydrated with Genteal eye drops, the ocular lens was positioned, and the image was viewed in real time. The optic nerve head was centered on the monitor, and an imaging scan was taken. Once the scan was completed and saved, the process was repeated for the second eye. The thickness of the retinal layers was then measured from the OCT images.

#### Immunolabeling and Histology

For immunolabeling experiments, rats were anesthetized using ketamine (100 mg/kg body weight) and xylazine (5 mg/kg body weight) delivered by intraperitoneal injection. Once anesthetized, the rat was secured on a foam block with pins and the thorax was cut through the rib cage to expose the heart. A cut was made to the right atrium and a 22-gauge needle was inserted into the left ventricle, and 0.1 M phosphate-buffered saline (PBS, pH 7.2) was pumped into the body for two minutes to remove all blood. Following exsanguination, 200 ml of 4% paraformaldehyde in 0.1 M PBS was pumped into the body over a 20-min period. After perfusion, the eyeballs were enucleated, the cornea was punctured, and the eyes were immersed in 4% paraformaldehyde in 0.1 M PBS or 0.1 M cacodylate buffer. The anterior segment was then removed, and the resulting eyecups were returned to the fixative for an additional 30 min to 4 h at 4 °C. Eyecups used for frozen sections were rinsed in PBS, cryoprotected in 30% sucrose, then embedded in an optimal cutting temperature medium (Sakura Tissue Tek; VWR, West Chester, PA) and frozen by placing the tissue on an aluminum plate half submerged in liquid nitrogen. Frozen sections (10–15 μm thickness) were prepared on a cryostat, collected onto Superfrost Plus slides (Fisher Scientific, Pittsburgh, PA), and stored at − 20 to − 30 °C until used.

Immunolabeling was performed as described previously [3, 43]. Frozen sections of fixed eyecups were rehydrated in Hank’s buffered salt solution (HBSS, pH 7.2), and non-specific labeling was blocked using a solution containing 2–10% normal goat serum plus 5% bovine serum albumin, 1% fish gelatin, and 0.1–0.5% Triton X-100 in HBSS (“blocker”). In some experiments, antigen retrieval in 10 mM citrate buffer (pH 6.0; heated to 95 °C) was performed for 30–60 min prior to blocking. The blocker was removed and primary antibody was applied overnight at room temperature. Sections were rinsed in HBSS and an appropriate secondary antibody was applied for 60–75 min at room temperature. In some experiments, peanut agglutinin (PNA) conjugated to AlexaFluor488 was added to the secondary antibody solution to identify cone photoreceptors. Sections were rinsed and mounted using Prolong Gold plus DAPI or Prolong Diamond plus DAPI (Molecular Probes/Thermo Scientific, Cat #P36935 or P36971, respectively) to retard photobleaching. Specificity of labeling methods was confirmed by omitting primary antibody or substituting normal rabbit serum for primary antibody. Specimens labeled using a primary antibody in combination with PNA showed no bleedthrough of signals between fluorescence channels.

Fluorescence microscopy was performed using an Olympus IX70 inverted fluorescence microscope (Olympus America) fitted with a QiCAM CCD camera controlled via QCapture software (QImaging). Figures were prepared using Photoshop software after calibration of the image scale. Adjustments to brightness and contrast were applied equally to all pixels in the image, if needed, to highlight specific labeling.

For histological evaluation of the retinal structure, the rats were perfused and eyecups were prepared as described above. The eyecups were embedded in paraffin, and sections of 5-μm thickness were cut along the vertical meridian through the optic nerve and stained with hematoxylin and eosin for light microscopic evaluation of the retinal structure. To assess photoreceptor loss, the thickness of the outer nuclear layer (ONL) was measured at 1-mm distances from the optic nerve to the inferior and superior ora serrata and plotted as a “spider” diagram.

#### Antibodies and Lectins

*Elongation of Very Long Chain Fatty Acids 4 (ELOVL4)*. Anti- ELOVL4 antibody was a rabbit polyclonal antiserum generated by our group [1]. Details of its production and specificity have been described previously [1, 44, 45]. This antibody recognizes a conserved C-terminal epitope and recognizes both the WT and W246G mutant forms of ELOVL4. Anti-ELOVL4 was used at a dilution of 1:300 to 1:500.

*Glial Fibrillary Acidic Protein (GFAP)*. (Millipore, Cat# MAB360. RRID:AB_2109815; mouse monoclonal, clone GA-5). Anti-GFAP recognizes a 51-kDa band on Western blots corresponding to GFAP from human glioma cells (U33CG/343MG) [46]. This antibody does not recognize vimentin. Anti-GFAP was used at a dilution of 1:500–1:2000.

*Glutamine synthetase (GS)*. (Millipore, Cat# MAB302, RRID:AB_2110656; mouse monoclonal, clone GS-6). Anti-GS was raised against full-length GS purified from sheep brain. Anti-GS recognizes a 45-kDa band on Western blots of sheep and rat brain [47]. Anti-GS was used at a dilution of 1:500.

*Peanut Agglutinin (PNA)*. Peanut agglutinin specifically labels the extracellular matrix surrounding cone outer segments [48]. PNA conjugated to AlexaFluor 488 (Molecular Probes/ThermoFisher Scientific Cat# L21409; RRID:AB_2315178) was used at a dilution of 1:20–1:50.

Secondary antibodies conjugated to AlexaFluor 568 were purchased from Molecular Probes/ThermoFisher Scientific and used at a dilution of 1:200–1:300 (goat anti-rabbit IgG AlexaFluor568 [Cat# A11036; RRID:AB_143011], goat anti-mouse IgG AlexaFluor568 [Cat# A11004; RRID:AB_2534072]). Normal sera were purchased from Jackson Immunoresearch (normal goat serum [Cat# 005-000-121; RRID:AB_2336990]; normal rabbit serum [Cat# 011-000-120; RRID:AB_2337123]).

#### Statistical Analysis

Statistical analysis was performed using one-way ANOVA with Tukey’s post hoc test using GraphPad Prism software (GraphPad, La Jolla, CA). Statistical significance was set to *p* < 0.05.

## Results

### Generation of Transgenic SCA34-KI Long-Evans Rats

We generated a novel line of Long-Evans rats using CRISPR/Cas9 by editing one copy of the wild-type (WT) *Elovl4* allele in the rat genome to 736T>G (p.W246G) to generate the SCA34-KI rats (Fig. 1). Genotyping and sequencing confirmed genomic editing of one copy of the WT *Elovl4* allele to the c. 736T>G SCA34 allele (Fig. 1b). Detailed Sanger and whole-genome sequencing of potential off-target sites confirmed no aberrant indels or insertions in the rat genome (Supplemental Figs. 1 and 2).

Heterozygous SCA34-KI F0 founders were crossed to WT Long-Evans rats to establish a breeding colony. SCA34-KI rats were viable and transmitted the SCA34 allele to the next generation to produce WT, heterozygote (HET), and homozygote (MUT) SCA34-KI pups in Mendelian ratios. Unlike STGD3 homozygous mice that die at birth due to dehydration [11–14], MUT SCA34-KI rats survived and were fertile.

**Fig. 2:**
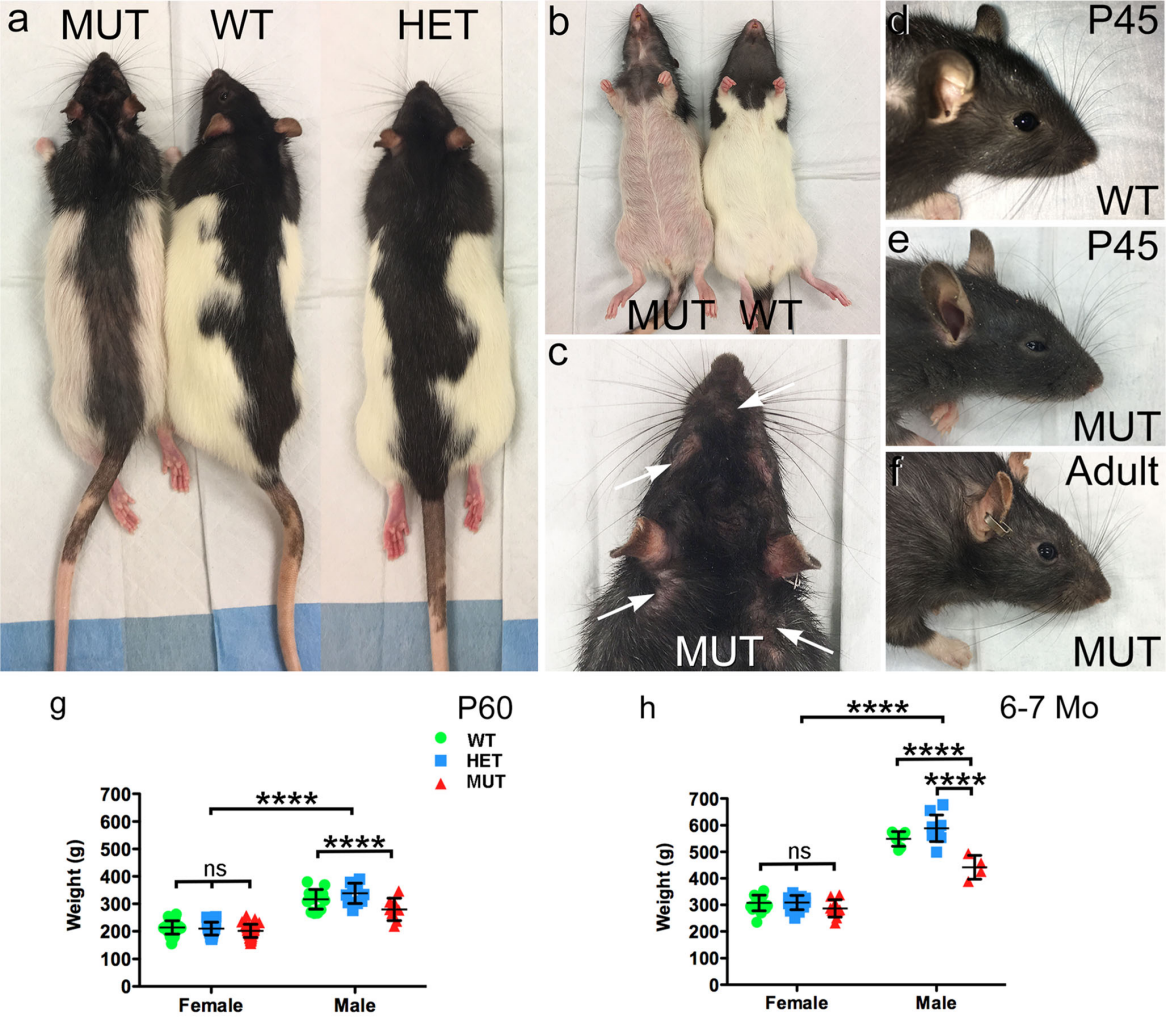
Gross physical phenotype of SCA34-KI rats. **a** Gross appearance of WT and HET SCA34-KI rats is similar. MUT SCA34-KI rats show hair loss and erythrokeratodermia variabilis (EKV). **b** Comparison of the underside of WT and MUT SCA34-KI rats showing marked hair loss and EKV. **c** Hair loss around the eyes, nose, and ears (arrows) on a MUT SCA34-KI rat. **d** WT rat pups show normal eyelids with full opening (P45 shown). **e** MUT SCA34-KI rats show stiff, swollen eyelids at early ages that open incompletely (P45 shown), which resolves by about P60. Hair loss around the eye is also common in MUT rats. **f** Adult MUT rats show complete opening of the lids. **g** Male rats of all genotypes were significantly larger than female rats by P60. Female rats showed no difference in weight among genotypes, but male MUT rats were significantly smaller than male WT or HET rats. (Females: *n* = 29 WT, 32 HET, 33 MUT; males: *n* = 14 WT, 11 HET, 10 MUT. Data shown as mean ± St. Dev. 2-way ANOVA + Bonferroni post hoc test (sex, genotype). *, *p* < 0.05; **, *p* < 0.01; ***, *p* < 0.001; ****, *p* < 0.0001). **h** Male rats of all genotypes were larger than female rats of all genotypes at 6–7 months of age. Female rats showed no difference in weight among genotypes, but male MUT rats were significantly smaller than male WT and HET rats. (Females: *n* = 17 WT, 14 HET, 10 MUT; males: *n* = 7 WT, 10 HET, 4 MUT. Data shown as mean ± St. Dev. 2-way ANOVA + Bonferroni post hoc test (sex, genotype). *, *p* < 0.05; **, *p* < 0.01; ***, *p* < 0.001; ****, *p* < 0.0001)

Although the MUT rats survived birth, there were differences in their appearance compared to WT and HET rats (Fig. 2a). MUT rats showed EKV with red, scaly skin and hair loss that was absent from WT and HET animals (Fig. 2a, b). EKV was especially prominent on the eyelids, and delayed eye opening by several days and caused swelling that persisted for several weeks (Fig. 2c–e). Eyelid swelling resolved between P60 and P90, permitting complete opening of the eyelid (Fig. 2f). As would be expected, body size differed significantly between age-matched male and female SCA34-KI rats. Female rats showed no differences in weight among genotypes at either P60 or 6–7 months of age (Fig. 2g–h). In contrast, male MUT rats were smaller than WT male rats by P60 and by 6–7 months of age were smaller than both WT and HET male rats.

### The W246G Mutation in ELOVL4 Selectively Affects VLC-SFA Synthesis

**Fig. 3:**
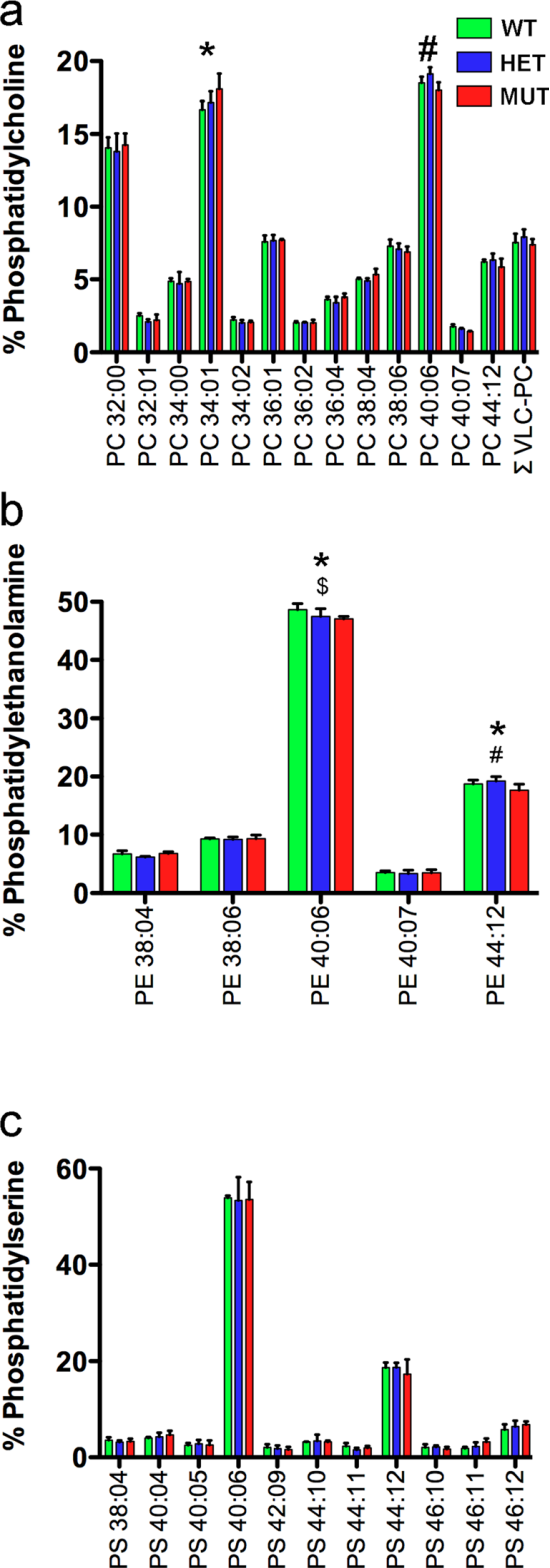
W246G ELOVL4 retains the ability to synthesize VLC-PUFA. Analysis of retinal glycerophospholipids from WT, HET, and MUT SCA34-KI rats shows that the W246G mutant form of ELOVL4 retains the capacity to synthesize VLC-PUFA at normal levels. No differences in total retinal levels of VLC-PUFA were present among WT, HET, and MUT rats. **a** VLC-PUFA were detected specifically in the phosphatidylcholine fraction (PC), but total VLC-PUFA levels showed no differences among WT, HET, and MUT rat retinas. However, significant differences were detected in non-VLC-FA (PC 34:01 and PC 40:06) among genotypes. **b** No VLC-PUFA were detected in the phosphatidylethanolamine (PE) fraction of WT, HET, or MUT rat retina. However, statistically significant differences were detected in PE 40:06 and PC 44:12. **c** No VLC-PUFA were detected in the phosphatidylserine (PS) fraction of WT, HET, or MUT rat retina. No significant differences were detected in any lipid species in the PS fraction. (Analysis by 1-way ANOVA with Tukey’s post hoc test. Data shown as mean ± St. Dev. *, MUT differs from WT; $, HET differs from WT; #, MUT differs from WT. See Supplemental Tables 1–3 for details)

Glycerophospholipid levels in WT, HET, and MUT rats were analyzed to assess the effect of the W246G mutation on the ability of ELOVL4 to catalyze the formation of VLC-PUFA in the retina (Fig. 3; details in Supplemental Tables 1–3). VLC-PUFA in the retina were found only in the phosphatidylcholine (PC) fraction (Fig. 3), as reported previously [15, 16]. There were no differences in total VLC-PUFA in PC (ΣVLC-PC) across genotypes, indicating that W246G ELOVL4 was able to catalyze VLC-PUFA formation normally. Some minor differences were found in shorter chain PUFA incorporated into PC and phosphatidylethanolamine (PE), but no differences in PUFA incorporated into phosphatidylserine (PS) were observed (Supplemental Tables 1–3).

**Fig. 4:**
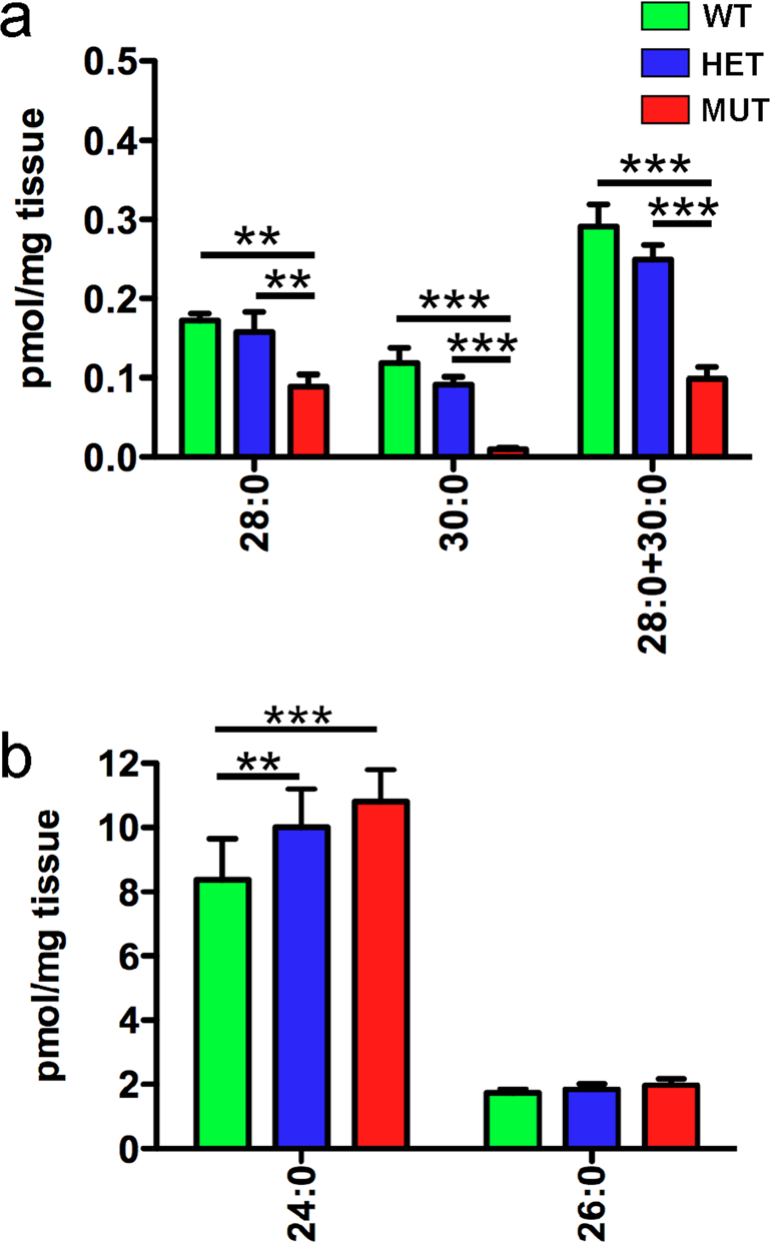
The W246G mutation in ELOVL4 impairs VLC-SFA synthesis. **a** Analysis of VLC-SFA in skin. Levels of VLC-SFA (28:0 and 30:0) and total VLC-SFA (28:0 + 30:0) were significantly reduced in the skin of MUT rats compared to WT and HET rats. **b** Levels of 26:0, the direct precursor for VLC-SFA synthesis, did not differ significantly across genotypes. However, levels of 24:0 were significantly elevated in the skin of HET and MUT rats compared to WT rats. (Data shown as mean ± St. Dev. Analysis by 1-way ANOVA with Tukey’s post hoc test. *, *p* < 0.05; **, *p* < 0.01; ***, *p* < 0.001. See Supplemental Table 4 for details)

ELOVL4 also catalyzes the formation of VLC-SFA in the skin [11–14], Meibomian gland [5], and central nervous system [3]. Because VLC-SFA are expressed at very low levels in central nervous tissue, making them difficult to detect [3], the ability of W246G ELOVL4 to catalyze the formation of VLC-SFA was assessed in skin, where they are much more abundant. The levels of VLC-SFA (28:0, 30:0, and 28:0 + 30:0) were significantly reduced in the skin of MUT rats compared to WT and HET rats (Fig. 4a; Supp. Table 4). Skin VLC-SFA levels did not differ significantly between WT and HET rats. Thus, the W246G mutation in ELOVL4 selectively impaired its ability to catalyze the formation of VLC-SFA. To better understand how the W246G ELOVL4 mutation affected synthesis of SFA levels overall, we also assessed the levels of 24:0 and 26:0, long-chain-SFA precursors needed for the synthesis of VLC-SFA. Consistent with inhibition of VLC-SFA formation, levels of 24:0, a precursor to VLC-SFA formation, were elevated in the skin of HET and MUT rats (Fig. 4b; Supp. Table 4). However, there were no significant differences in levels of 26:0, the direct precursor of 28:0.

### The W246G Form of ELOVL4 Does Not Induce Retinal Degeneration

**Fig. 5:**
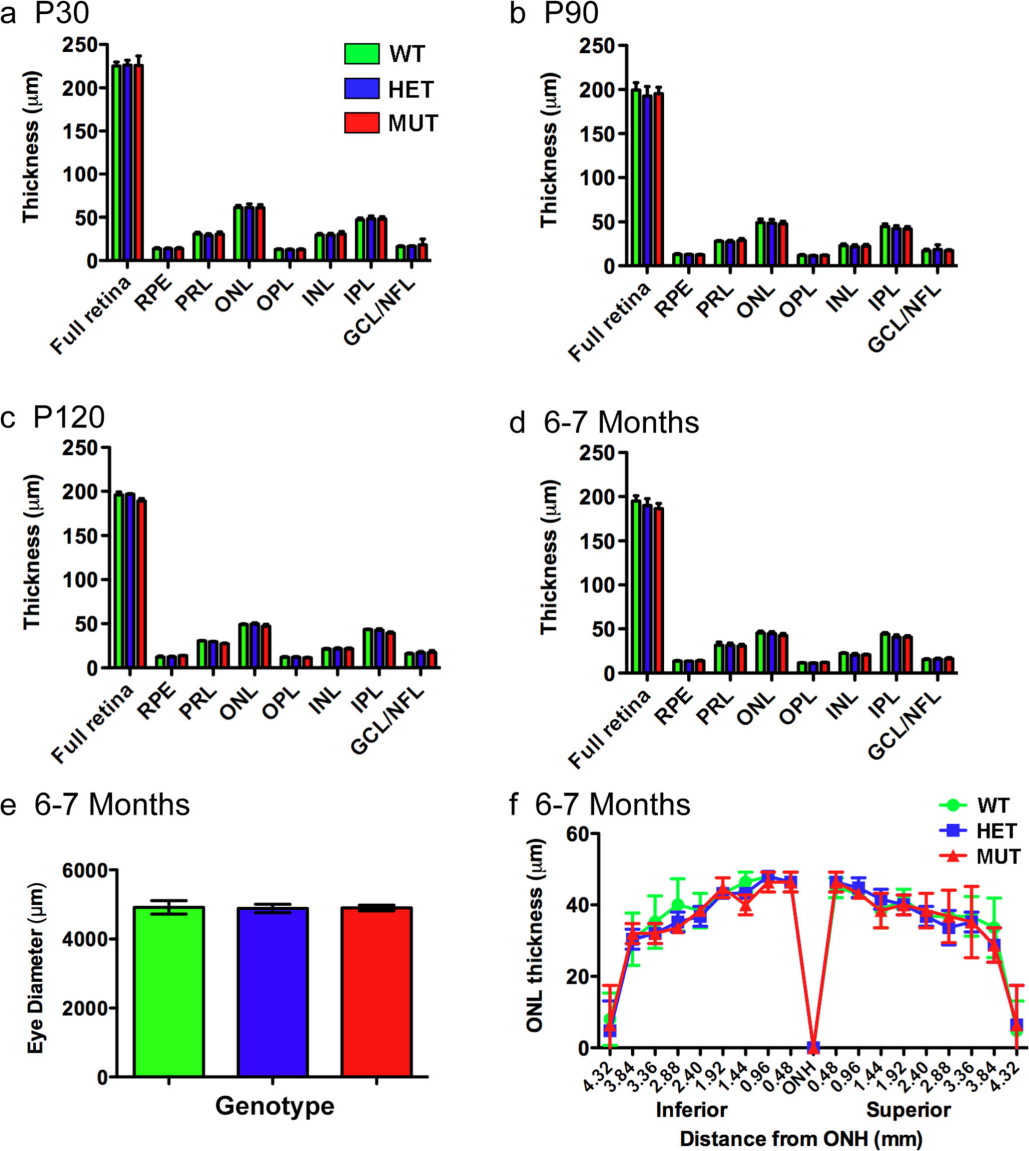
Retinal layering is normal and photoreceptors are preserved in the SCA34-KI rat retinas to at least 6–7 months. **a**–**d** Optical coherence tomography (OCT) analysis of WT, HET, and MUT SCA34 rat retinas from P30 to 6–7 months of age showed no differences in thickness of any retinal layer at any time point. (Data shown as mean ± St Dev. Analysis by 1-way ANOVA with Tukey’s post hoc test. P30: 12 WT, 13 HET, 16 MUT. P90: 9 WT, 14 HET, 10 MUT. P120: 2 WT, 2 HET, 5 MUT. 6–7 months: 7 WT, 11 HET, 7 MUT). **e** Eye diameter in 6–7-month-old rats also is unaffected by the W246G mutation in ELOVL4. (One-way ANOVA with Tukey’s post hoc test. Data shown as mean ± St Dev. *n* = 3 WT, 3 HET, 3 MUT). **f** Histological analysis of outer nuclear layer thickness shows no differences among WT, HET, and MUT rats along the superior to inferior axis at 6–7 months of age, indicating no significant photoreceptor degeneration. (One-way ANOVA with Tukey’s post hoc test. Data shown as mean ± St Dev. *n* = 3 WT, 3 HET, 3 MUT). PRL, photoreceptor layer; ONL, outer nuclear layer; OPL, outer plexiform layer; INL, inner nuclear layer; IPL, inner plexiform layer; GCL, ganglion cell layer

To assess the presence or absence of retinal degeneration, retinal integrity was assessed using optical coherence tomography (OCT), histological, and immunolabeling approaches (Fig. 5). OCT analysis revealed no differences in total retinal thickness or in the thickness of individual retinal layers among WT, HET, and MUT rats between P30 and 6–7 months of age. Histological assessment of outer nuclear layer (ONL) thickness in the retinas of WT, HET, and MUT rats at 6–7 months of age showed no differences among genotypes, indicating that no significant loss of photoreceptors was present by this time. Analysis of eye diameter at 6–7 months of age also showed no differences among genotypes, indicating that the W246G mutation in ELOVL4 did not affect the size of the eye.

**Fig. 6:**
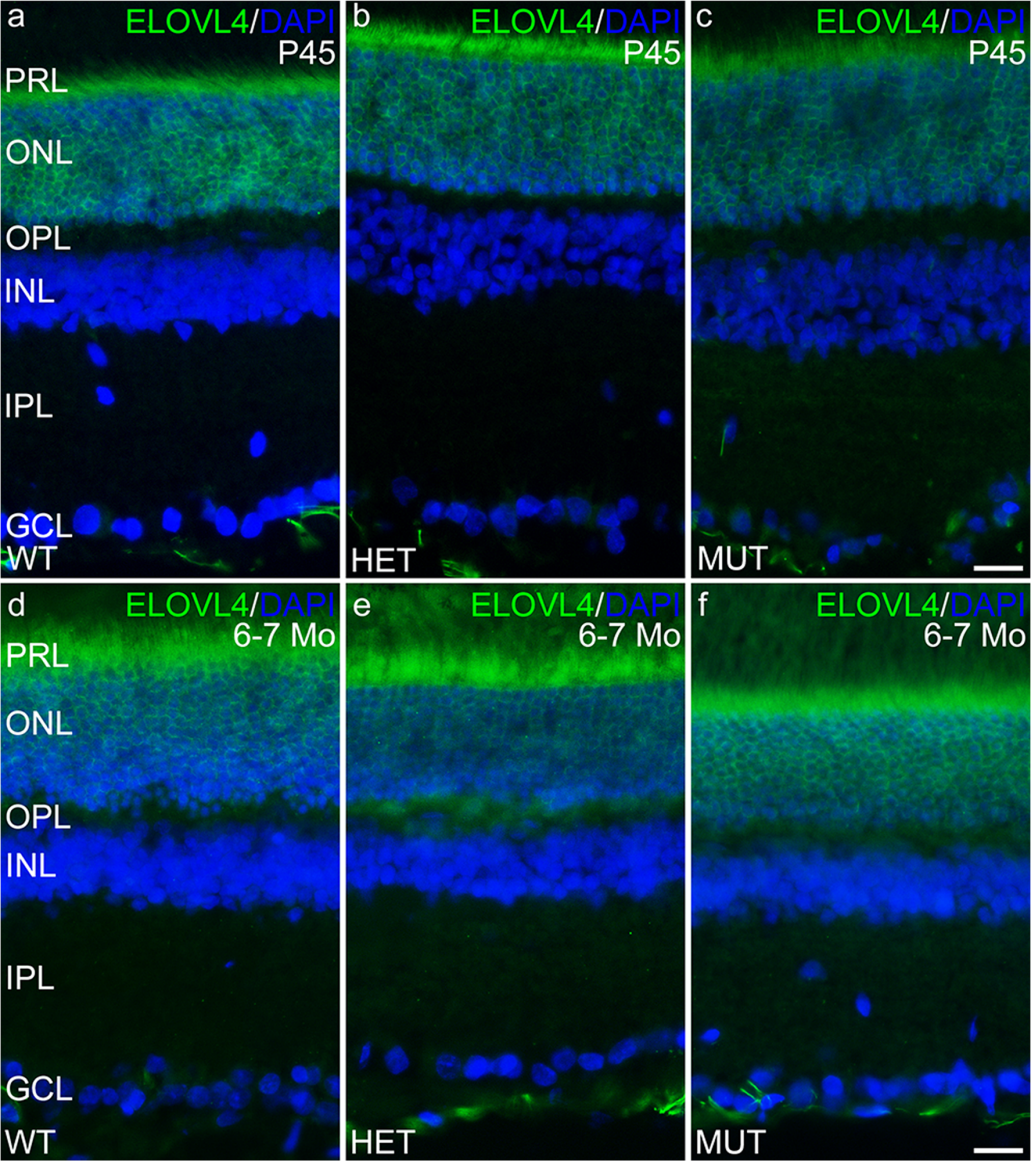
The distribution of ELOVL4 is normal in the SCA34-KI rat retina. **a**–**c** ELOVL4 immunolabeling in the retina of P45 WT, HET, and MUT rats is present in the photoreceptor cells, as appropriate. The ELOVL4 antibody recognizes an epitope that is conserved in WT and W246G mutant ELOVL4. **d**–**f** Normal distribution of ELOVL4 immunolabeling is preserved in the photoreceptors in WT, HET, and MUT rats at 6–7 months of age (6–7 Mo). PRL, photoreceptor layer; ONL, outer nuclear layer; OPL, outer plexiform layer; INL, inner nuclear layer; IPL, inner plexiform layer; GCL, ganglion cell layer. Scale bars = 50 μm

**Fig. 7:**
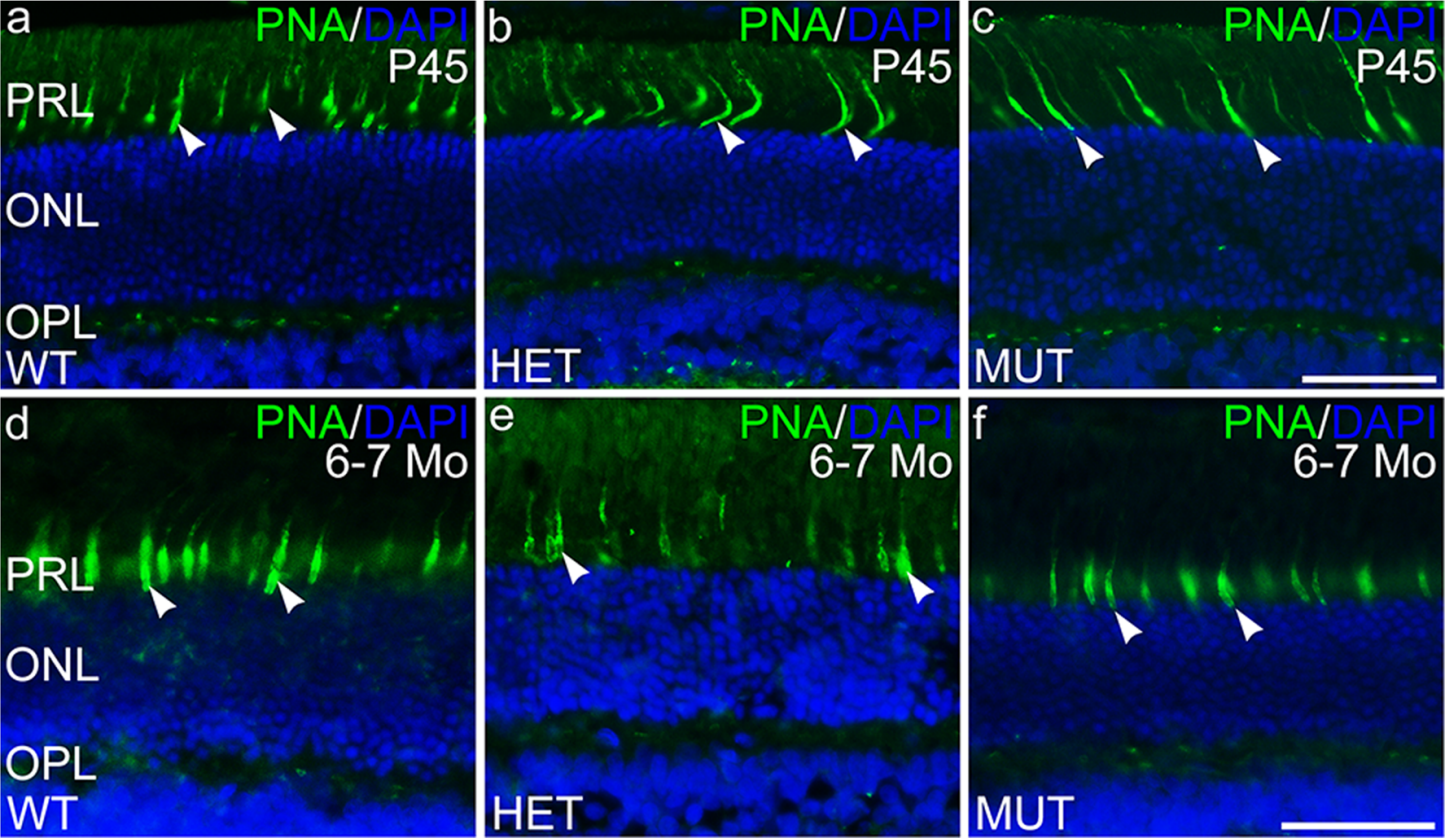
Cone cells are preserved in the SCA34-KI rat retina. **a**–**c** Labeling of cone outer segment sheaths (arrowheads) is normal in the retina of P45 WT, HET, and MUT rats. **d**–**f** Normal PNA labeling of cone outer segment sheaths is preserved in the retinas of 6–7 month-old (6–7 Mo) WT, HET, and MUT rats. PRL, photoreceptor layer; ONL, outer nuclear layer; OPL, outer plexiform layer; INL, inner nuclear layer; IPL, inner plexiform layer; GCL, ganglion cell layer. Scale bars = 50 μm

**Fig. 8:**
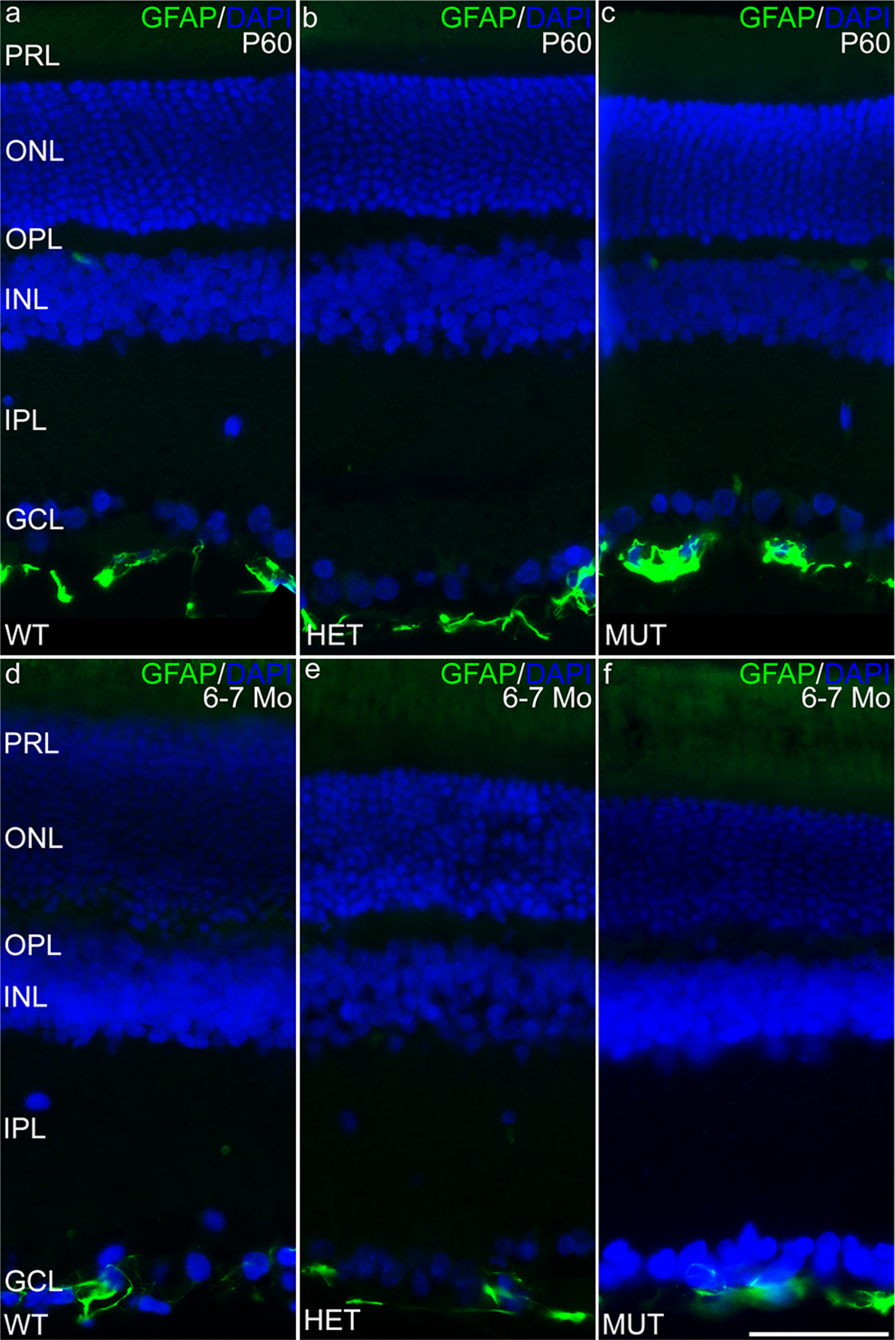
Müller cells in the SCA34-KI rat retina are not reactive. **a**–**c** Müller cells in the retina of P45 WT, HET, and MUT rats show little labeling for glial fibrillary acidic protein (GFAP) as is typical of Müller cells in the healthy retina. Labeling in blood vessels (bv) is non-specific. **d**–**f** Müller cells in the retina of 6–7-month-old (6–7 Mo) WT, HET, and MUT rats also show little labeling for GFAP, indicating the absence of glial reactivity associated with retinal degeneration. PRL, photoreceptor layer; ONL, outer nuclear layer; OPL, outer plexiform layer; INL, inner nuclear layer; IPL, inner plexiform layer; GCL, ganglion cell layer. Scale bars = 50 μm

Immunolabeling for ELOVL4 and other markers for photoreceptors, Müller cells, and neurodegeneration was performed to further assess potential changes in the SCA34-KI rat retina with age (P45 to 6–7 months of age). Labeling for ELOVL4 in the WT, HET, and MUT retinas was normal (Fig. 6), with high levels of labeling in photoreceptor cell bodies in the ONL. Labeling for PNA also was normal, confirming that cones and their synapses were maintained in retinas of all genotypes (Fig. 7). Müller cells showed no elevated labeling for GFAP, a sensitive marker of reactive gliosis and retinal degeneration (Fig. 8). Labeling for glutamine synthetase, a marker for Müller cells, showed normal Müller cell morphology and distribution in retinas of all genotypes (Supplemental Fig. 3).

### The W246G SCA34 Mutation in ELOVL4 Impairs Retinal Function

Mutations in *ELOVL4* that cause STGD3 profoundly impair retinal light responses and cause early-onset macular degeneration in STGD3 patients [26, 27, 31–33, 36]. In contrast, human SCA34 patients typically show no retinal disease. To determine whether the W246G mutation in ELOVL4 affected retinal function, electroretinogram (ERG) recordings were made from WT, HET, and MUT rats. Rats of all three genotypes showed rod- and cone-driven ERG responses at P30 and P60, but eyelid swelling in MUT rats partially obstructed the pupil, confounding interpretation of results at these time points. Therefore, ERG studies focused on P90, P120, and 6–7 month time points, after eyelid swelling resolved.

**Fig. 9:**
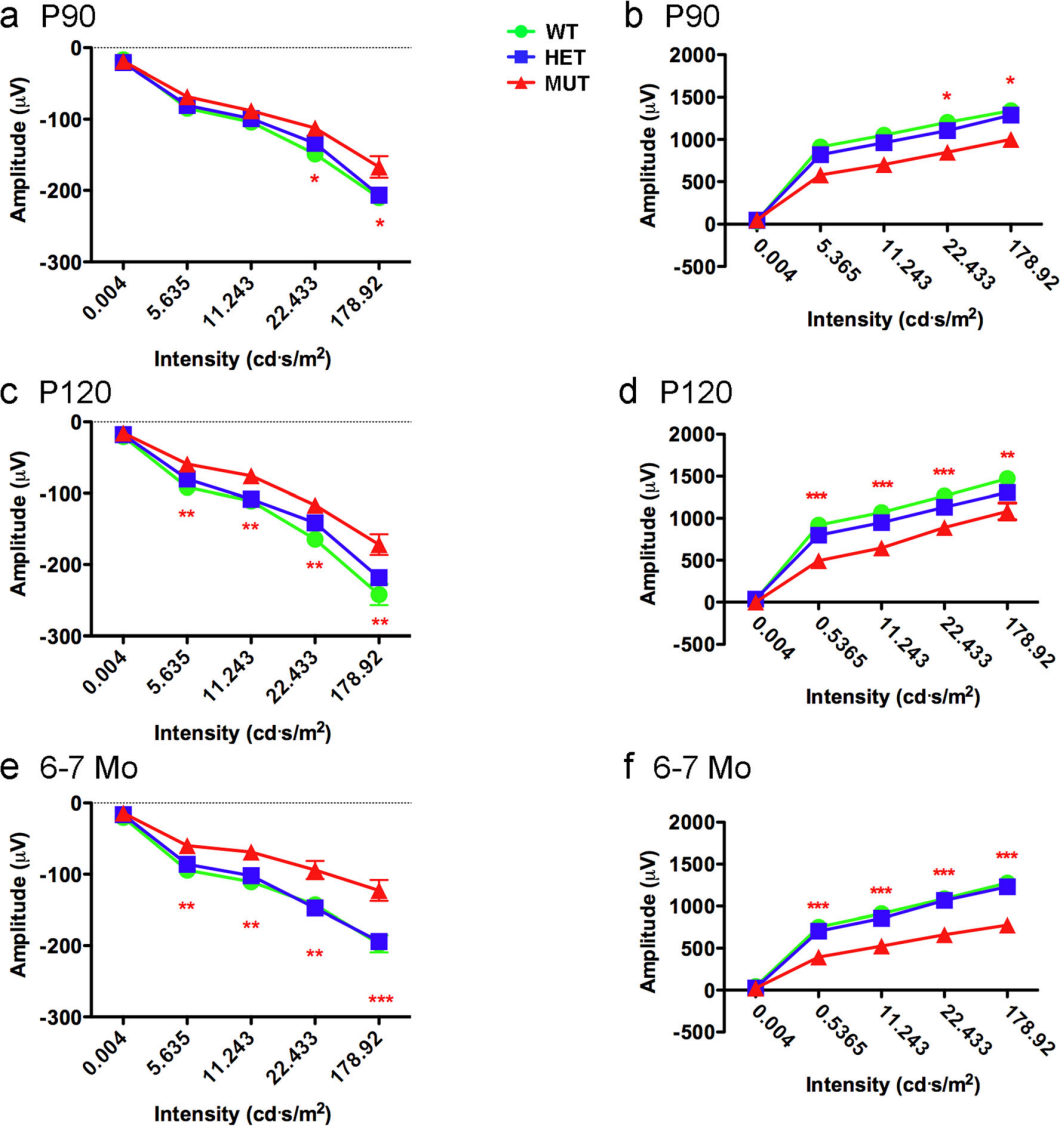
Effects of the W246G mutant form of ELOVL4 on the scotopic ERG. Homozygous inheritance of the W246G form of ELOVL4 reduces scotopic a-wave and b-wave ERG responses. **a**, **c**, **e** Scotopic ERG a-wave amplitude for WT, HET, and MUT rats at P90, P120, and 6–7 months of age. **b**, **d**, **f** Scotopic ERG b-wave amplitude for WT, HET, and MUT rats at P90, P120, and 6–7 months of age. (Data shown as mean ± SEM. One-way ANOVA with Tukey’s post hoc test. Asterisks indicate statistical significance at *p* < 0.05 (*), *p* < 0.01 (**), and *p* < 0.001 (***). P90: 14 WT, 14 HET, 17 MUT. P120: 13 WT, 15 HET, 15 MUT. 6–7 Mo: 9 WT, 14 HET, 7 MUT)

**Fig. 10:**
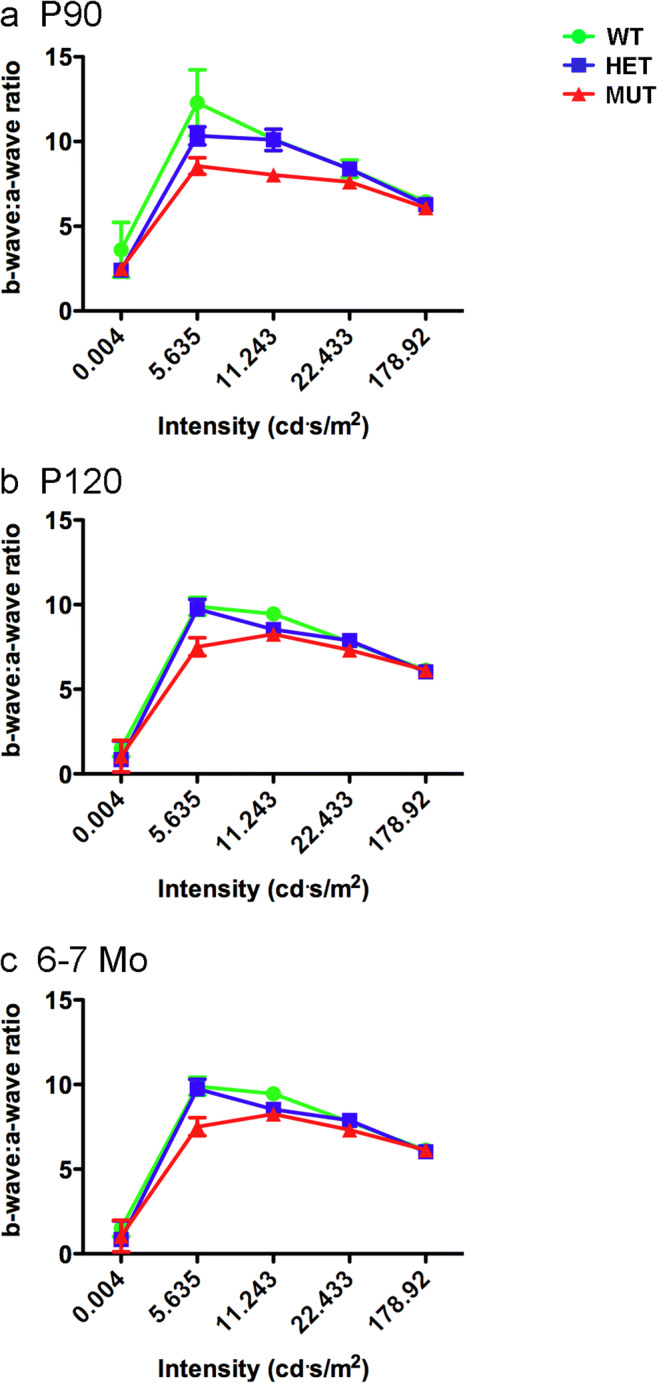
Ratio of scotopic b-wave to a-wave amplitude. The scotopic b-wave to a-wave ratio in MUT rats shows a consistent reduction compared to the b-wave to a-wave ratio of age-matched HET and WT rats, suggesting that amplification of rod signals transmitted to the inner retina is reduced in MUT rats. (Data shown as mean ± SEM. One-way ANOVA with Tukey’s post hoc test. Asterisks indicate statistical significance at *p* < 0.05 (*), *p* < 0.01 (**), and *p* < 0.001 (***). P90: 14 WT, 14 HET, 17 MUT. P120: 13 WT, 15 HET, 15 MUT. 6–7 Mo: 9 WT, 14 HET, 7 MUT)

Analysis of rod-driven, scotopic ERG responses revealed that the scotopic a-wave amplitude of MUT rats was reduced compared to that of WT and HET rats (Fig. 9a–e). At P90, the scotopic a-wave showed significantly reduced amplitude only at relatively high flash intensities, but significant differences in amplitude were present at all but the lowest flash intensity at P120 and 6–7 months. The scotopic b-wave of MUT rats showed a similar reduction in amplitude compared to that of WT and HET rats (Fig. 9b–f). There were no differences in scotopic a-wave or b-wave amplitudes between WT and HET rats at any flash intensity or age. To assess whether synaptic transmission of signals from rods to rod bipolar cells was affected, the scotopic b-wave to a-wave ratio was assessed (Fig. 10). The b-wave to a-wave ratio of MUT rats consistently was slightly reduced compared to that of age-matched WT and HET rats, suggesting that MUT rats may show a small reduction in amplification of rod signals transmitted to the inner retina. Latency of the scotopic a-wave and time to peak of the b-wave were not substantially affected, although a significant difference in a-wave latency was present at P90 at the lowest flash intensity tested (Supplemental Fig. 4).

**Fig. 11:**
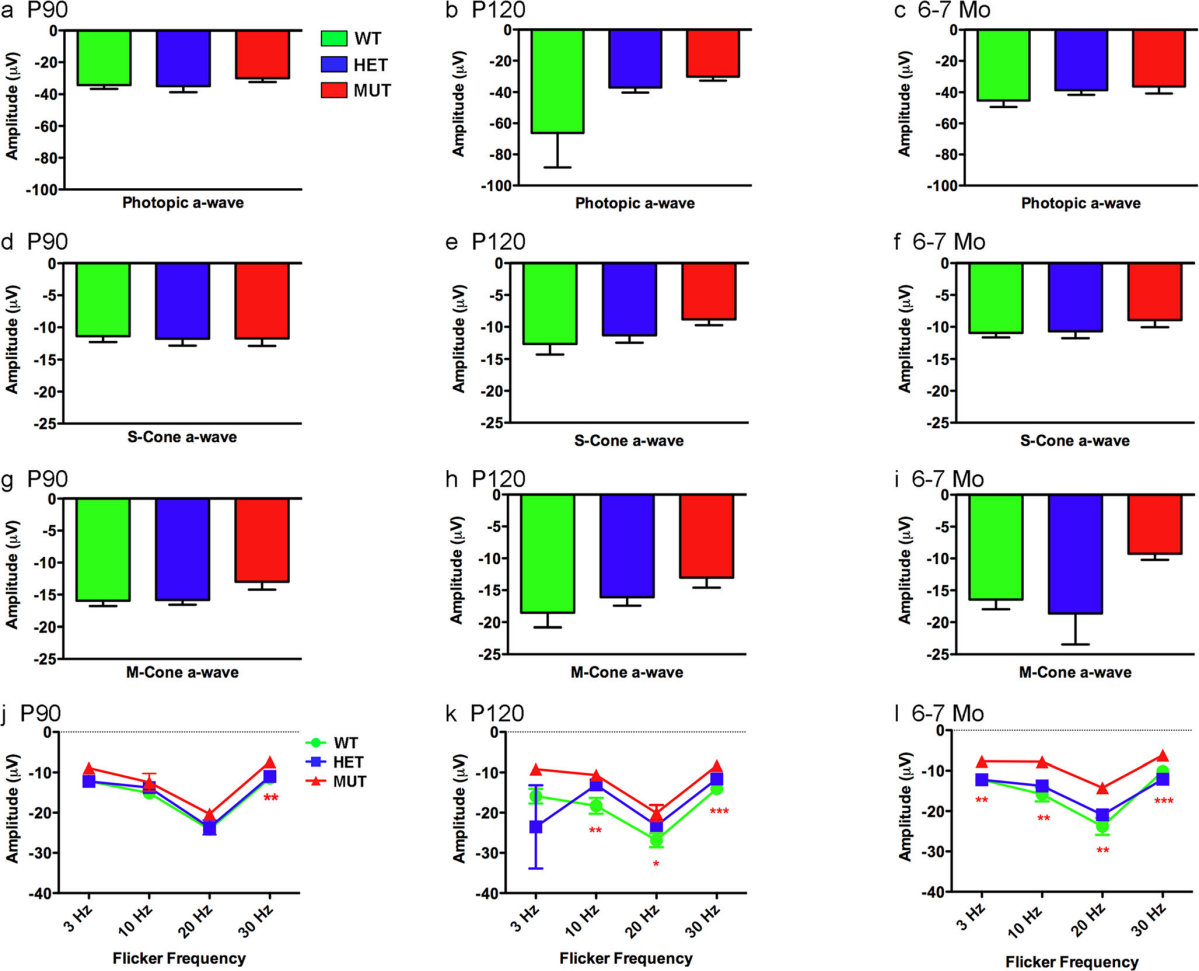
Effects of the W246G mutant form of ELOVL4 on the photopic ERG a-wave. Homozygous inheritance of the W246G form of ELOVL4 reduces cone-driven ERG a-wave responses. **a**, **b**, **c** Photopic ERG a-wave amplitude for WT, HET, and MUT rats at P90, P120, and 6–7 months of age. **d**, **e**, **f** S-cone-driven ERG a-wave amplitude for WT, HET, and MUT rats at P90, P120, and 6–7 months of age. **g**, **h**, **i** M-cone-driven ERG a-wave amplitude for wild-type, heterozygote, and homozygote SCA34-KI rats at P90, P120, and 6–7 months of age. **j**, **k**, **l** Cone-driven flicker ERG a-wave amplitude for WT, HET, and MUT rats at P90, P120, and 6–7 months of age. (Data shown as mean ± SEM. One-way ANOVA with Tukey’s post hoc test. Asterisks indicate statistical significance at *p* < 0.05 (*), *p* < 0.01 (**), and *p* < 0.001 (***). P90: 14 WT, 14 HET, 17 MUT. P120: 13 WT, 15 HET, 15 MUT. 6–7 Mo: 9 WT, 14 HET, 7 MUT)

The W246G mutation in ELOVL4 also affected cone-driven ERG responses. MUT rats showed slightly reduced amplitudes for the photopic a-wave, and S-cone- and M-cone-driven a-waves compared to WT and HET at P90, P120, and 6–7 months; however, these differences were not statistically significant (Fig. 11a–i). Cone-driven a-wave latencies showed no differences across genotypes (Supplemental Fig. 5). In contrast, the flicker ERG a-wave of MUT rats differed significantly from that of WT and HET rats (Fig. 11j–l), indicating that the W246G mutation in ELOVL4 affected the cone-driven flicker responses at the level of the cones themselves. The flicker ERG a-wave showed no significant differences between WT and HET rats. Cone-driven flicker ERG a-wave latencies showed no differences across genotypes (Supplemental Fig. 6).

**Fig. 12:**
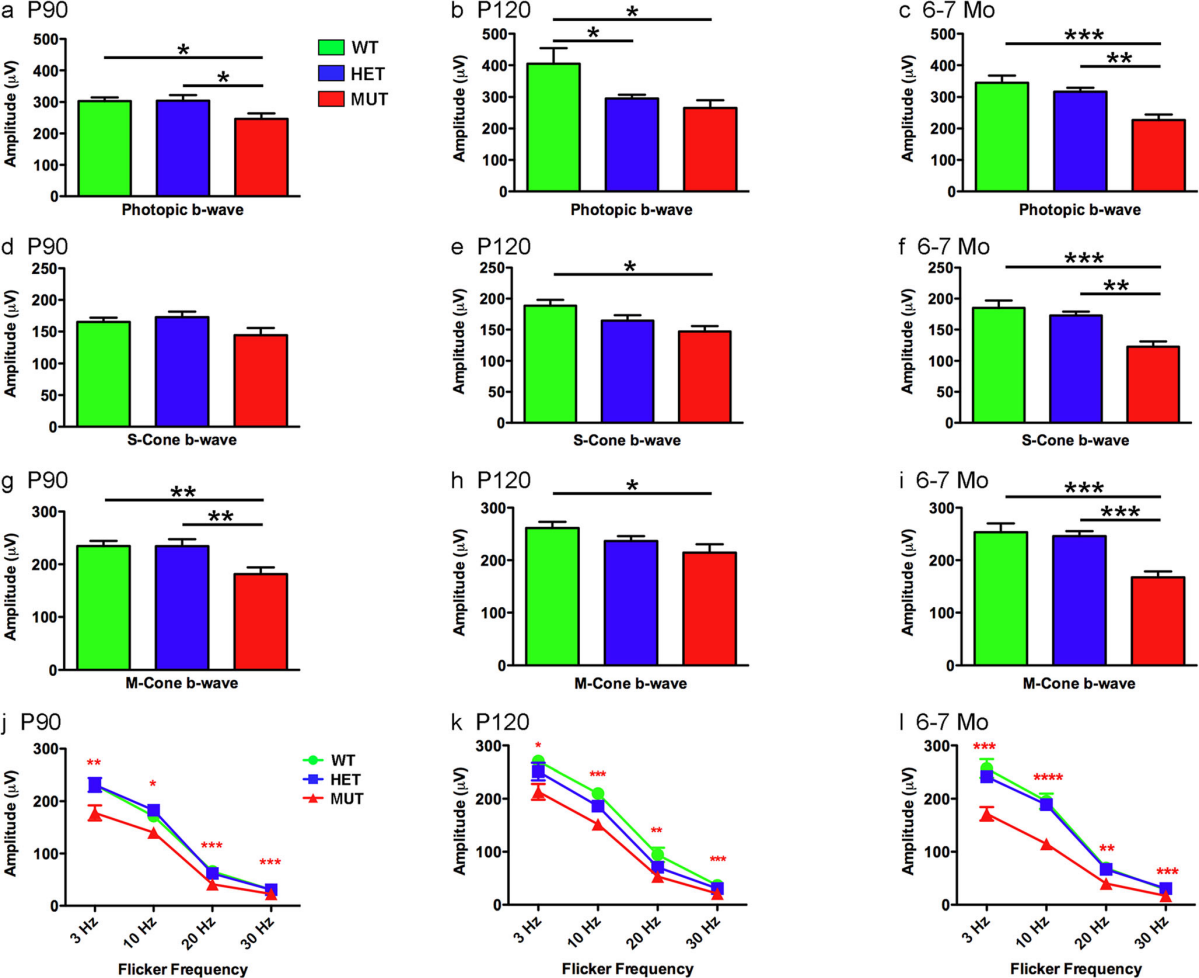
Effects of the W246G mutant form of ELOVL4 on the photopic ERG b-wave. Homozygous inheritance of the W246G form of ELOVL4 reduces cone-driven ERG b-wave responses. **a**, **b**, **c** Photopic ERG b-wave amplitude for WT, HET, and MUT rats at P90, P120, and 6–7 months of age. **d**, **e**, **f** S-cone-driven ERG b-wave amplitude for WT, HET, and MUT rats at P90, P120, and 6–7 months of age. **g**, **h**, **i** M-cone-driven ERG b-wave amplitude for WT, HET, and MUT rats at P90, P120, and 6–7 months of age. **j**, **k**, **l** Cone-driven flicker ERG b-wave amplitude for WT, HET, and MUT rats at P90, P120, and 6–7 months of age. (Data shown as mean ± SEM. One-way ANOVA with Tukey’s post hoc test. Asterisks indicate statistical significance at *p* < 0.05 (*), *p* < 0.01 (**), and *p* < 0.001 (***). P90: 14 WT, 14 HET, 17 MUT. P120: 13 WT, 15 HET, 15 MUT. 6–7 Mo: 9 WT, 14 HET, 7 MUT)

Cone-driven b-wave responses were more strongly affected by the W246G mutation in ELOVL4. The photopic b-wave amplitude of MUT rats was significantly reduced compared to that of WT rats at all ages tested (Fig. 12a–c). Similarly, the S-cone, M-cone, and flicker ERG b-wave amplitudes of MUT rats also were reduced significantly compared to those of WT rats (Fig. 12d–l). In some cases, additional differences in cone-driven b-wave amplitudes were noted among MUT, HET, and WT control rats (Fig. 12a–i). Cone-driven b-wave time-to-peak measures showed no differences across genotypes at any age (Supplemental Figs. 6 and 7).

## Discussion

Several different mutations in *ELOVL4* cause age-related SCA34 characterized by prominent progressive cerebellar atrophy and ataxia in patients. Here, we report successful application of the CRISPR/Cas9 approach to generate the first rat model of human SCA34 caused by the c. 736T>G (p.W246G) mutation in the human *ELOVL4* as reported by Ozaki et al. [35]. The F0 heterozygous rats were viable and transmitted the 736T>G (p.W246G) mutation to the next generation as determined by genotyping and sequencing. Unlike mice in which the homozygous expression of the STGD3 mutant ELOVL4 or global deletion of ELOVL4 causes neonatal lethality [11–14], homozygous W246G MUT rats survive birth and thrive to adulthood, although the males are smaller in size compared to age-matched WT and HET rats. Both Sanger sequencing and whole gDNA sequencing confirm no off-target effects in the HET and MUT rats. Retinal ELOVL4 expression in all three genotypes is similar, suggesting that the W246G mutation did not affect retinal *Elovl4* expression and protein synthesis.

The physiological, biochemical, and anatomical studies presented herein indicate that the W246G mutation in ELOVL4 that causes SCA34, a neurodegenerative disease that strikes the cerebellum and can be associated with EKV in the skin, also affects retinal function in the absence of retinal degeneration. This is the first report to characterize functional retinal deficits associated with SCA34-causing mutations in ELOVL4. Functional and anatomical studies showed that homozygous W246G ELOVL4 mutation depressed retinal light responses in the absence of neurodegeneration. Biochemical studies indicate that the W246G ELOVL4 mutation impaired production of VLC-SFA, but spared synthesis of VLC-PUFA.

The retinal and skin phenotypes of HET rats are similar to those of human SCA34 patients heterozygous for the W246G ELOVL4 mutation [35]. HET rats, like human SCA34 patients with the W246G ELOVL4 mutation, do not show severe EKV [35], although EKV is present in SCA34 patients with other *ELOVL4* mutations [28–30]. Similarly, HET rats show retinal responses comparable to WT rats, consistent with the reported absence of functional retinal deficits in human SCA34 patients [35]. No human patients homozygous for any SCA34-causing *ELOVL4* mutation have been reported. Speculation is that this results from these mutant alleles being very rare in the population and/or that homozygous inheritance of these alleles would be embryonically lethal. Therefore, the birth and survival of MUT rats were unexpected and presented a unique opportunity to further explore ELOVL4 function.

ELOVL4 is essential for synthesis of both VLC-PUFA and VLC-SFA [1–3]. In the retina, VLC-PUFA are the main product and are incorporated into PC and concentrated in the outer segment membranes [15–18]. VLC-PUFA levels in WT, HET, and MUT rat retinas did not differ, indicating that W246G ELOVL4 supported normal VLC-PUFA biosynthesis. Some small differences in PUFA with shorter chain lengths, which are not produced directly by ELOVL4, were detected in SCA34-KI rats. In contrast to VLC-PUFA, the homozygous W246G mutation in ELOVL4 impaired synthesis of VLC-SFA (28:0 and 30:0) by 50% or more in the skin of MUT rats compared to WT and HET rats. Synthesis of 30:0 was more severely affected than 28:0. No statistical differences in VLC-SFA levels were detected between WT and HET rats. Thus, the W246G mutation in ELOVL4 selectively impairs VLC-SFA synthesis while leaving VLC-PUFA overtly unaffected.

Mutations in ELOVL4 that cause STGD3 all have deletion of the C-terminal ER retention motif that leads to mislocalization of the enzyme from the ER, reduced ELOVL4 protein levels, and a dominant negative effect on VLC-PUFA biosynthesis [49, 50]. In contrast, W246G ELOVL4 retains the ER retention motif in the C-terminal and would be directed to the ER membranes. However, in the homozygous state, the W246G ELOVL4 mutation seems to affect VLC-SFA biosynthesis selectively. In contrast, VLC-PUFA synthesis was spared in HET and MUT rats, while VLC-SFA synthesis was impaired in the skin of MUT rats, indicating that the W246G ELOVL4 mutation affects VLC-SFA synthesis while retinal VLC-PUFA synthesis was spared. The molecular basis for this selective impairment of VLC-SFA synthesis is unknown, but one possibility is that the W246G mutation alters the structure in or around the active site such that access of the long-chain-SFA precursor (26:0) to the site is impaired, whereas access of the VLC-PUFA precursors (26:Xn3 and 26:Yn6) is not. Additionally, ELOVL4 could have differential protein-protein interactions in the retina that lead to differential tissue-specific effects on VLC-SFA and VLC-PUFA synthesis. Importantly, the effect of the W246G mutation on the molecular structure of the ELOVL4 protein remains unknown.

Human SCA34 patients heterozygous for the W246G ELOVL4 mutation have no reported clinical deficits in retinal function [35], although no ERG results were presented for these patients. Similarly, the rod- and cone-driven ERG responses of HET and WT rats were comparable, consistent with our lipid analyses that showed no differences in VLC-fatty acid levels between these genotypes. In contrast, MUT rats showed a clear functional retinal deficit. MUT rats showed significant reductions in scotopic ERG a-wave and b-wave amplitudes compared to WT and HET rats. The effect of the W246G *ELOVL4* mutation on the photopic ERG was less pronounced. The photopic a-wave of MUT rats differed from that of WT and HET rats only for the flicker ERG, but several significant differences in photopic ERG b-wave measures were found between the MUT rats and WT and HET rats. These deficits appeared by 90 days of age and persisted until at least 6–7 months of age, the oldest age tested in this study.

Recently, a study of 10 affected members of an American family with a novel heterozygous *ELOVL4* mutation (I171T) that results in spinocerebellar ataxia and retinitis pigmentosa was reported [37]. This mutation causes a late-onset spinocerebellar ataxia similar to other SCA34-causing *ELOVL4* mutations [28–30, 35]. This SCA is characterized by average onset in the 5th decade, often accompanied by dysarthria and handwriting changes, but no autonomic dysfunction or dermatologic disease [37]. In contrast to other previously known SCA34-causing *ELOVL4* mutations, which showed no retinal degeneration [28–30, 35], the I171T ELOVL4 mutation was associated with retinitis pigmentosa, a degenerative retinal disease, in 4 of the 10 family members examined (three males and one female).

The W246G *ELOVL4* mutation affected the ERG b-wave, which is dependent on signal transmission from photoreceptors to second-order bipolar cells, more strongly than the a-wave, which reflects the light-driven responses of the photoreceptors themselves. The finding that the scotopic ERG b-wave to a-wave ratio of MUT rats was consistently lower than that of age-matched WT and HET rats also suggests a reduction of signal amplification across the photoreceptor to the bipolar cell synapse in MUT rats. A recent study in our laboratory in mice homozygous for the 5-base-pair STGD3 deletion in ELOVL4 showed that slices of MUT hippocampus had aberrant presynaptic neurotransmitter release kinetics and seizure activity [3]. Re-supply of exogenous VLC-SFA in MUT cultured hippocampal neurons restored presynaptic release kinetics to normal levels. A role for ELOVL4 and its VLC-SFA products in photoreceptor transmission is supported by the finding of VLC-SFA in retinal sphingolipids [7] and that mouse photoreceptors with the 5-base-pair STGD3 deletion in ELOVL4 have smaller synaptic vesicles and reduced b-wave amplitude compared to WT mice [44, 51].

The functional deficits in the ERG of MUT rats are unlikely to arise from neurodegeneration. Anatomical analyses of retinal layer thickness and photoreceptor numbers revealed no indications of neurodegeneration in the retina of HET or MUT rats out to 6–7 months of age. Immunolabeling studies confirmed that photoreceptors expressed ELOVL4 appropriately across all genotypes and appeared structurally normal. Müller glial cells, which show striking reactivity and elevated levels of GFAP in the presence of degeneration, showed no reactivity in the HET or MUT retina. Our lipid analyses showed that VLC-PUFA were present in PC at normal levels in the HET and MUT retinas, which would maintain the retrograde elovanoid feedback signaling from the retinal pigmented epithelium to the photoreceptors needed to promote photoreceptor survival [52], consistent with our studies showing an absence of neurodegeneration in the HET and MUT retinas.

Diseases arising from *ELOVL4* mutations likely represent a continuum arising from differential effects of the various mutations on ELOVL4’s function in the synthesis of VLC-SFA and VLC-PUFA. Our current studies suggest that *ELOVL4* mutations that selectively impair VLC-SFA synthesis compromise synaptic function in the CNS and the barrier function of the skin. The W246G *ELOVL4* mutation examined here selectively impaired synthesis of VLC-SFA, with no significant effect on VLC-PUFA synthesis, leading to compromised ERG responses in MUT rats in the absence of retinal degeneration. These results are consistent with the aberrant presynaptic release and seizures induced by VLC-SFA deficiency due to homozygous knock-in of the 5-base-pair STGD3 mutation [3]. The appearance of EKV in only MUT rats is also consistent with the VLC-SFA deficiency associated with homozygous W246G ELOVL4 mutation. Heterozygous *ELOVL4* mutations that cause SCA34 with EKV [28–30] presumably compromise VLC-SFA synthesis more severely than the W246G ELOVL4 mutation. The novel I171T ELOVL4 mutation that causes SCA accompanied by retinitis pigmentosa in some patients [37] would be predicted to impair synthesis of VLC-SFA and also VLC-PUFA to some degree. Heterozygous *Elovl4* mutations that cause STGD3, a severe juvenile-onset photoreceptor degeneration [27, 32, 36], compromise VLC-PUFA biosynthesis [49, 50]. STGD3 patients have no other reported CNS or skin disease, suggesting that heterozygous STGD3 mutations may not alter VLC-SFA synthesis substantially, although complete deletion of *Elovl4* expression in mice is neonatal lethal. Homozygous inheritance of STGD3-causing *ELOVL4* mutations has not been reported in humans, but mice homozygous for the 5-bp STGD3 mutation (but kept alive by transgenic expression of *Elovl4* in their skin) have disrupted synaptic function and severe seizures at P18 [3], indicating that homozygous inheritance of STGD3 alleles compromises synthesis of both VLC-PUFA and VLC-SFA. Finally, ELOVL4 neuro-ichthyotic syndrome, the most severe disease associated with *ELOVL4* mutations, is characterized by seizures, spasticity, intellectual disability, ichthyosis, and early death. This syndrome arises from homozygous *ELOVL4* mutations that cause early truncation of the protein [25, 34], presumably eliminating all VLC-SFA and VLC-PUFA synthesis.

Based on our enzyme activity studies and a previous study showing that VLC-SFA are enriched in synaptic vesicles and regulate presynaptic release kinetics in the brain [3], the most likely explanation for impaired ERG responses in MUT rats is that reduced production of VLC-SFA in photoreceptors impairs neurotransmission from photoreceptors to the second-order neurons. The possibility that ELOVL4 is involved in the biosynthesis or regulation of other, as yet unidentified, lipid products cannot be dismissed. The role of ELOVL4 in the production of VLC-SFA was not recognized until a decade after its role in VLC-PUFA biosynthesis was first identified [1]. The possibility that VLC-SFA might serve some unrecognized signaling function cannot be dismissed. Only recently has it been recognized that VLC-PUFA incorporated into phosphatidylcholine in photoreceptor outer segment membranes are metabolized by the retinal pigmented epithelium to form elovanoid signaling molecules that provide feedback signals that are essential for photoreceptor survival [52]. Further mechanistic studies will be needed to resolve these questions.

Taken together, these findings provide additional support for the notion that ELOVL4 and its VLC-SFA and VLC-PUFA products are critical to different aspects of neural function. The W246G mutation in ELOVL4 appears to selectively reduce, but not eliminate, production of VLC-SFA, leading to impaired transmission from photoreceptors to the inner retina, while maintaining normal VLC-PUFA biosynthesis needed for survival of photoreceptors.

## Electronic Supplementary Material

Supplemental Figure 1 **Sanger sequencing confirms appropriate gene editing.** Sequencing from the 5′-3′ primer direction (left to right on the figure), Sanger DNA sequencing of WT, HET, and MUT rat DNA sequences confirms the single point mutation c.736 T > G in the rat *Elovl4* genome. Box and arrows indicate site of gene editing. **a.** MUT. **b.** HET. **c**. WT. (PNG 1558 kb)

High resolution image (TIF 5700 kb)

Supplemental Figure 2 **Whole genome sequence analysis.** Reading from the 3′-5 primer direction (right to left on the figure), whole genome sequencing of WT and MUT rats confirms knockin of the 736 T > G, p.W246G mutant Elovl4 without any major off target effects in MUT rats (MUT). The box highlights the position of the 736 T > G mutation. Each gray bar represents a NextGen sequence. Colored bases differ from the WT sequence. Bases matching the WT are shown in gray to highlight only mutant bases. Examples of whole genome sequencing from two WT (WT 1 and WT2) and two MUT (MUT1 and MUT2) rats are shown. (PNG 385 kb)

High resolution image (TIF 1852 kb)

Supplemental Figure 3 **Glutamine synthetase labeling (GS, green) and Müller cell morphology is normal in the WT, HET, and MUT SCA34-KI rat retina. a-c.** P45. **d-f.** 6–7 months of age. Labeling associated with the blood vessels in the retina is non-specific. Retina counterstained with DAPI (blue) to show nuclear layers. PRL, photoreceptor layer; ONL, outer nuclear layer; OPL, outer plexiform layer; INL, inner nuclear layer; IPL, inner plexiform layer; GCL, ganglion cell layer. Scale bars = 50 μm for each row. (PNG 1546 kb)

High resolution image (TIF 2002 kb)

Supplemental Figure 4 Analysis of scotopic a-wave latency (**a,c,e**) and b-wave time-to-peak (**b,d,f**) at P90, P120, and P180 in WT, HET and MUT SCA34-KI rats. (Data shown as mean ± SEM. One-way ANOVA with Tukey’s post-hoc test. Asterisk indicates statistical significance at *p* < 0.05 for WT response compared to HET and MUT response at P90 in panel A. P90: 14 WT, 14 HET, 17 MUT. P120: 13 WT, 15 HET, 15 MUT. 6–7 Mo: 9 WT, 14 HET, 7 MUT). (PNG 397 kb)

High resolution image (TIF 566 kb)

Supplemental Figure 5 Analysis of photopic (S + M-cone)-driven a-wave latency (**a,d,g**), S-cone-driven a-wave latency (**b,e,h**), and M-cone-driven a-wave latency (**c,f,i**) at P90, P120, and 6–7 Mo in WT, HET, and MUT SCA34-KI rats. (Data shown as mean ± SEM. One-way ANOVA with Tukey’s post-hoc test. P90: 14 WT, 14 HET, 17 MUT. P120: 13 WT, 15 HET, 15 MUT. 6–7 Mo: 9 WT, 14 HET, 7 MUT). (PNG 253 kb)

High resolution image (TIF 474 kb)

Supplemental Figure 6 Analysis of flicker ERG a-wave latency (**a,c,e**), b-wave time to peak (**b,d,f**) at P90, P120, and 6–7 Mo in WT, HET, and MUT rats. (Data shown as mean ± SEM. One-way ANOVA with Tukey’s post-hoc test. P90: 14 WT, 14 HET, 17 MUT. P120: 13 WT, 15 HET, 15 MUT. 6–7 Mo: 9 WT, 14 HET, 7 MUT). (PNG 334 kb)

High resolution image (TIF 503 kb)

Supplemental Figure 7 Analysis of photopic (S + M-cone)-driven b-wave time-to-peak (**a,d,g**), S-cone-driven b-wave time-to-peak (**b,e,h**), and M-cone-driven b-wave time-to-peak (**c,g,i**) at P90, P120, and 6–7 Mo in WT, HET, and MUT SCA34-KI rats. (Data shown as mean ± SEM. One-way ANOVA with Tukey’s post-hoc test. P90: 14 WT, 14 HET, 17 MUT. P120: 13 WT, 15 HET, 15 MUT. 6–7 Mo: 9 WT, 14 HET, 7 MUT). (PNG 280 kb)

High resolution image (TIF 561 kb)

ESM 1 (PDF 56 kb)

ESM 2 (PDF 53 kb)

ESM 3 (PDF 49 kb)

ESM 4 (PDF 42 kb)

## Data Availability

The raw data will not be deposited in a public repository due to the large size of the image files and the complexity of annotating the large volume of individual files.

## References

[1] Agbaga MP, Brush RS, Mandal MN, Henry K, Elliott MH, Anderson RE (2008). Role of Stargardt-3 macular dystrophy protein (ELOVL4) in the biosynthesis of very long chain fatty acids. Proc Natl Acad Sci U S A.

[2] Agbaga MP, Mandal MN, Anderson RE (2010). Retinal very long-chain PUFAs: new insights from studies on ELOVL4 protein. J Lipid Res.

[3] Hopiavuori BR, Deak F, Wilkerson JL, Brush RS, Rocha-Hopiavuori NA, Hopiavuori AR, Ozan KG, Sullivan MT, Wren JD, Georgescu C, Szweda L, Awasthi V, Towner R, Sherry DM, Anderson RE, Agbaga MP (2018). Homozygous expression of mutant ELOVL4 leads to seizures and death in a novel animal model of very long-chain fatty acid deficiency. Mol Neurobiol.

[4] Mandal MN, Ambasudhan R, Wong PW, Gage PJ, Sieving PA, Ayyagari R (2004). Characterization of mouse orthologue of ELOVL4: genomic organization and spatial and temporal expression. Genomics.

[5] McMahon A, Lu H, Butovich IA (2014). A role for ELOVL4 in the mouse meibomian gland and sebocyte cell biology. Invest Ophthalmol Vis Sci.

[6] Poulos A, Johnson DW, Beckman K, White IG, Easton C (1987). Occurrence of unusual molecular species of sphingomyelin containing 28-34-carbon polyenoic fatty acids in ram spermatozoa. Biochem J.

[7] Brush RS, Tran JT, Henry KR, McClellan ME, Elliott MH, Mandal MN (2010). Retinal sphingolipids and their very-long-chain fatty acid-containing species. Invest Ophthalmol Vis Sci.

[8] Hopiavuori BR, Agbaga MP, Brush RS, Sullivan MT, Sonntag WE, Anderson RE (2017). Regional changes in CNS and retinal glycerophospholipid profiles with age: a molecular blueprint. J Lipid Res.

[9] Poulos A, Sharp P, Johnson D, Easton C (1988). The occurrence of polyenoic very long chain fatty acids with greater than 32 carbon atoms in molecular species of phosphatidylcholine in normal and peroxisome-deficient (Zellweger's syndrome) brain. Biochem J.

[10] Robinson BS, Johnson DW, Poulos A (1990). Unique molecular species of phosphatidylcholine containing very-long-chain (C24-C38) polyenoic fatty acids in rat brain. Biochem J.

[11] Cameron DJ, Tong Z, Yang Z, Kaminoh J, Kamiyah S, Chen H, Zeng J, Chen Y, Luo L, Zhang K (2007). Essential role of Elovl4 in very long chain fatty acid synthesis, skin permeability barrier function, and neonatal survival. Int J Biol Sci.

[12] Li W, Sandhoff R, Kono M, Zerfas P, Hoffmann V, Ding BC, Proia RL, Deng CX (2007). Depletion of ceramides with very long chain fatty acids causes defective skin permeability barrier function, and neonatal lethality in ELOVL4 deficient mice. Int J Biol Sci.

[13] McMahon A, Butovich IA, Mata NL, Klein M, Ritter R, Richardson J, Birch DG, Edwards AO, Kedzierski W (2007). Retinal pathology and skin barrier defect in mice carrying a Stargardt disease-3 mutation in elongase of very long chain fatty acids-4. Mol Vis.

[14] Vasireddy V, Uchida Y, Salem N, Kim SY, Mandal MN, Reddy GB, Bodepudi R, Alderson NL, Brown JC, Hama H, Dlugosz A, Elias PM, Holleran WM, Ayyagari R (2007). Loss of functional ELOVL4 depletes very long-chain fatty acids (> or =C28) and the unique omega-O-acylceramides in skin leading to neonatal death. Hum Mol Genet.

[15] Agbaga MP, Merriman DK, Brush RS, Lydic TA, Conley SM, Naash MI, Jackson S, Woods AS, Reid GE, Busik JV, Anderson RE (2018). Differential composition of DHA and very-long-chain PUFAs in rod and cone photoreceptors. J Lipid Res.

[16] Aveldano MI (1987). A novel group of very long chain polyenoic fatty acids in dipolyunsaturated phosphatidylcholines from vertebrate retina. J Biol Chem.

[17] Aveldano MI, Sprecher H (1987). Very long chain (C24 to C36) polyenoic fatty acids of the n-3 and n-6 series in dipolyunsaturated phosphatidylcholines from bovine retina. J Biol Chem.

[18] Rotstein NP, Pennacchiotti GL, Sprecher H, Aveldano MI (1996). Active synthesis of C24:5, n-3 fatty acid in retina. Biochem J.

[19] Rabionet M, Bayerle A, Jennemann R, Heid H, Fuchser J, Marsching C, Porubsky S, Bolenz C, Guillou F, Grone HJ, Gorgas K, Sandhoff R (2015). Male meiotic cytokinesis requires ceramide synthase 3-dependent sphingolipids with unique membrane anchors. Hum Mol Genet.

[20] Rabionet M, van der Spoel AC, Chuang CC, von Tumpling-Radosta B, Litjens M, Bouwmeester D, Hellbusch CC, Korner C, Wiegandt H, Gorgas K, Platt FM, Grone HJ, Sandhoff R (2008). Male germ cells require polyenoic sphingolipids with complex glycosylation for completion of meiosis: a link to ceramide synthase-3. J Biol Chem.

[21] Santiago Valtierra FX, Penalva DA, Luquez JM, Furland NE, Vasquez C, Reyes JG, Aveldano MI, Oresti GM (2018). Elovl4 and Fa2h expression during rat spermatogenesis: A link to the very-long-chain PUFAs typical of germ cell sphingolipids. J Lipid Res.

[22] Agbaga MP (2016). Different mutations in ELOVL4 affect very long chain fatty acid biosynthesis to cause variable neurological disorders in humans. Adv Exp Med Biol.

[23] Deak F, Anderson RE, Fessler JL, Sherry DM (2019). Novel cellular functions of very long chain-fatty acids: insight from ELOVL4 mutations. Front Cell Neurosci.

[24] Walden CM, Sandhoff R, Chuang CC, Yildiz Y, Butters TD, Dwek RA, Platt FM, van der Spoel AC (2007). Accumulation of glucosylceramide in murine testis, caused by inhibition of beta-glucosidase 2: Implications for spermatogenesis. J Biol Chem.

[25] Aldahmesh MA, Mohamed JY, Alkuraya HS, Verma IC, Puri RD, Alaiya AA, Rizzo WB, Alkuraya FS (2011). Recessive mutations in ELOVL4 cause ichthyosis, intellectual disability, and spastic quadriplegia. Am J Hum Genet.

[26] Bardak H, Gunay M, Ercalik Y, Bardak Y, Ozbas H, Bagci O, Ayata A, Sonmez M, Alagoz C (2016) Analysis of ELOVL4 and PRPH2 genes in Turkish Stargardt disease patients. Genet Mol Res, 15. 10.4238/gmr15048774 27813578

[27] Bernstein PS, Tammur J, Singh N, Hutchinson A, Dixon M, Pappas CM, Zabriskie NA, Zhang K, Petrukhin K, Leppert M, Allikmets R (2001). Diverse macular dystrophy phenotype caused by a novel complex mutation in the ELOVL4 gene. Invest Ophthalmol Vis Sci.

[28] Bourassa CV, Raskin S, Serafini S, Teive HA, Dion PA, Rouleau GA (2015). A new ELOVL4 mutation in a case of spinocerebellar ataxia with erythrokeratodermia. JAMA Neurol.

[29] Bourque PR, Warman-Chardon J, Lelli DA, LaBerge L, Kirshen C, Bradshaw SH, Hartley T, Boycott KM (2018). Novel ELOVL4 mutation associated with erythrokeratodermia and spinocerebellar ataxia (SCA 34). Neurol Genet.

[30] Cadieux-Dion M, Turcotte-Gauthier M, Noreau A, Martin C, Meloche C, Gravel M, Drouin CA, Rouleau GA, Nguyen DK, Cossette P (2014). Expanding the clinical phenotype associated with ELOVL4 mutation: study of a large French-Canadian family with autosomal dominant spinocerebellar ataxia and erythrokeratodermia. JAMA Neurol.

[31] Donato L, Scimone C, Rinaldi C, Aragona P, Briuglia S, D'Ascola A, D'Angelo R, Sidoti A (2018). Stargardt phenotype associated with two ELOVL4 promoter variants and ELOVL4 downregulation: new possible perspective to etiopathogenesis?. Invest Ophthalmol Vis Sci.

[32] Edwards AO, Donoso LA, Ritter R (2001). A novel gene for autosomal dominant Stargardt-like macular dystrophy with homology to the SUR4 protein family. Invest Ophthalmol Vis Sci.

[33] Maugeri A, Meire F, Hoyng CB, Vink C, Van Regemorter N, Karan G, Yang Z, Cremers FP, Zhang K (2004). A novel mutation in the ELOVL4 gene causes autosomal dominant Stargardt-like macular dystrophy. Invest Ophthalmol Vis Sci.

[34] Mir H, Raza SI, Touseef M, Memon MM, Khan MN, Jaffar S, Ahmad W (2014). A novel recessive mutation in the gene ELOVL4 causes a neuro-ichthyotic disorder with variable expressivity. BMC Med Genet.

[35] Ozaki K, Doi H, Mitsui J, Sato N, Iikuni Y, Majima T, Yamane K, Irioka T, Ishiura H, Doi K, Morishita S, Higashi M, Sekiguchi T, Koyama K, Ueda N, Miura Y, Miyatake S, Matsumoto N, Yokota T, Tanaka F, Tsuji S, Mizusawa H, Ishikawa K (2015). A novel mutation in ELOVL4 leading to spinocerebellar ataxia (SCA) with the hot cross bun sign but lacking erythrokeratodermia: a broadened spectrum of SCA34. JAMA Neurol.

[36] Zhang K, Kniazeva M, Han M, Li W, Yu Z, Yang Z, Li Y, Metzker ML, Allikmets R, Zack DJ, Kakuk LE, Lagali PS, Wong PW, MacDonald IM, Sieving PA, Figueroa DJ, Austin CP, Gould RJ, Ayyagari R, Petrukhin K (2001). A 5-bp deletion in ELOVL4 is associated with two related forms of autosomal dominant macular dystrophy. Nat Genet.

[37] Xiao C, Binkley EM, Rexach J, Knightjohnson A, Khemani P, Fogel BL, Das S, Stone EM, Gomez CM (2019). A family with spinocerebellar ataxia and retinitis pigmentosa attributed to an ELOVL4 mutation. Neurol Genet.

[38] Huang M, Verbeek DS (2019). Why do so many genetic insults lead to Purkinje cell degeneration and spinocerebellar ataxia?. Neurosci Lett.

[39] Paulson HL, Shakkottai VG, Clark HB, Orr HT (2017). Polyglutamine spinocerebellar ataxias - from genes to potential treatments. Nat Rev Neurosci.

[40] Folch J, Lees M, Sloane Stanley GH (1957). A simple method for the isolation and purification of total lipides from animal tissues. J Biol Chem.

[41] Martin RE, Elliott MH, Brush RS, Anderson RE (2005). Detailed characterization of the lipid composition of detergent-resistant membranes from photoreceptor rod outer segment membranes. Invest Ophthalmol Vis Sci.

[42] Busik JV, Reid GE, Lydic TA (2009). Global analysis of retina lipids by complementary precursor ion and neutral loss mode tandem mass spectrometry. Methods Mol Biol.

[43] Sherry DM, Hopiavuori BR, Stiles MA, Rahman NS, Ozan KG, Deak F, Agbaga MP, Anderson RE (2017). Distribution of ELOVL4 in the developing and adult mouse brain. Front Neuroanat.

[44] Bennett LD, Hopiavuori BR, Brush RS, Chan M, Van Hook MJ, Thoreson WB, Anderson RE (2014). Examination of VLC-PUFA-deficient photoreceptor terminals. Invest Ophthalmol Vis Sci.

[45] Marchette LD, Sherry DM, Brush RS, Chan M, Wen Y, Wang J, Ash JD, Anderson RE, Mandal NA (2014). Very long chain polyunsaturated fatty acids and rod cell structure and function. Adv Exp Med Biol.

[46] Debus E, Weber K, Osborn M (1983). Monoclonal antibodies specific for glial fibrillary acidic (GFA) protein and for each of the neurofilament triplet polypeptides. Differentiation.

[47] Kentroti S, Baker R, Lee K, Bruce C, Vernadakis A (1991). Platelet-activating factor increases glutamine synthetase activity in early and late passage C-6 glioma cells. J Neurosci Res.

[48] Blanks JC, Johnson LV (1984). Specific binding of peanut lectin to a class of retinal photoreceptor cells. A species comparison. Invest Ophthalmol Vis Sci.

[49] Logan S, Agbaga MP, Chan MD, Brush RS, Anderson RE (2014). Endoplasmic reticulum microenvironment and conserved histidines govern ELOVL4 fatty acid elongase activity. J Lipid Res.

[50] Logan S, Agbaga MP, Chan MD, Kabir N, Mandal NA, Brush RS, Anderson RE (2013). Deciphering mutant ELOVL4 activity in autosomal-dominant Stargardt macular dystrophy. Proc Natl Acad Sci U S A.

[51] Bennett LD, Brush RS, Chan M, Lydic TA, Reese K, Reid GE, Busik JV, Elliott MH, Anderson RE (2014). Effect of reduced retinal VLC-PUFA on rod and cone photoreceptors. Invest Ophthalmol Vis Sci.

[52] Jun B, Mukherjee PK, Asatryan A, Kautzmann MA, Heap J, Gordon WC, Bhattacharjee S, Yang R, Petasis NA, Bazan NG (2017). Elovanoids are novel cell-specific lipid mediators necessary for neuroprotective signaling for photoreceptor cell integrity. Sci Rep.

