# Supplementary figures and images for: The Elovl4 Spinocerebellar Ataxia-34 Mutation 736T>G (p.W246G) Impairs Retinal Function in the Absence of Photoreceptor Degeneration
Source: Mol Neurobiol. 2020 Aug 11;57(11):4735–53. doi: 10.1007/s12035-020-02052-8 (PMC7515967; doi:10.1007/s12035-020-02052-8)

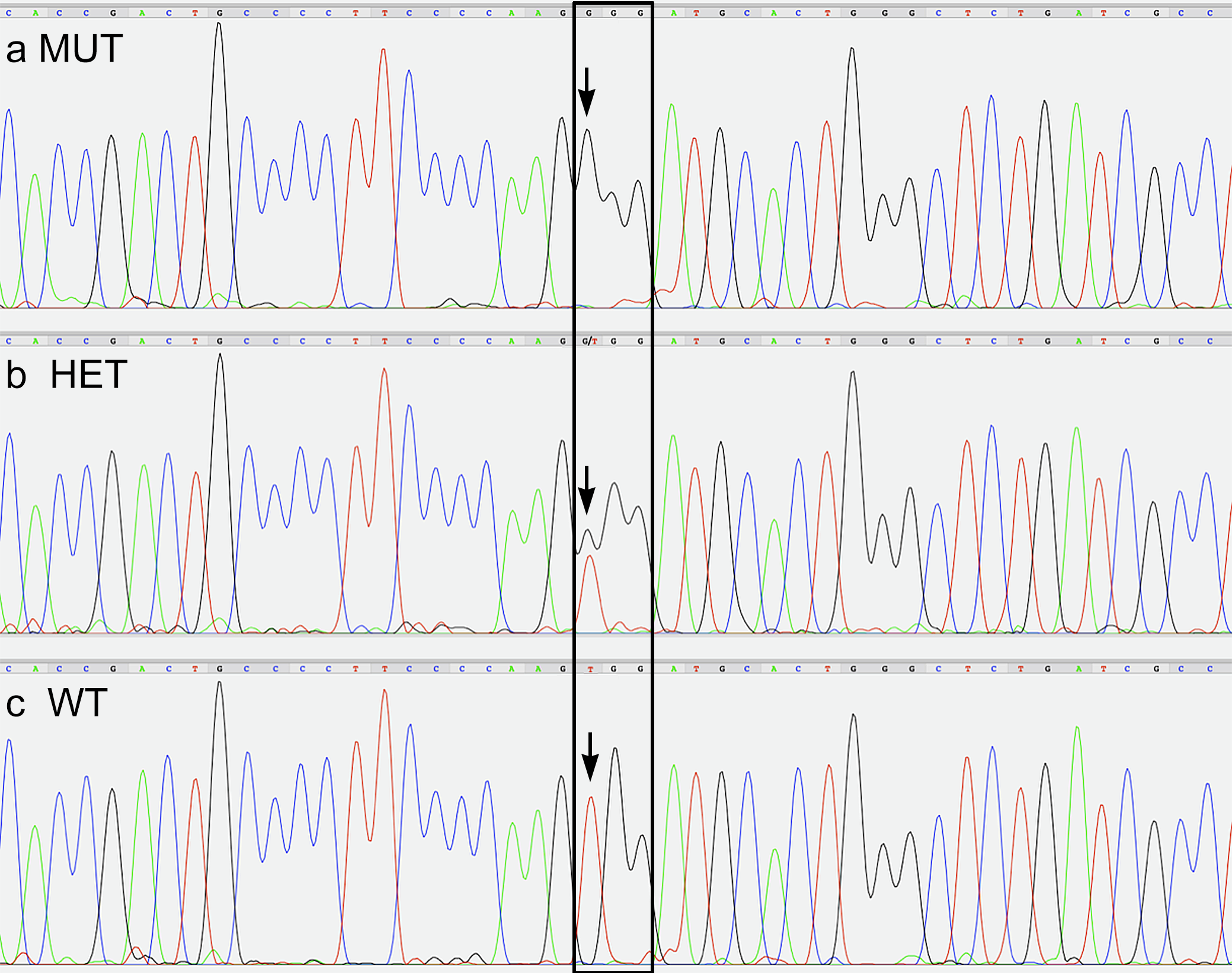

Supplement: Supplementary file 1 — Sanger sequencing confirms appropriate gene editing. Sequencing from the 5′-3′ primer direction (left to right on the figure), Sanger DNA sequencing of WT, HET, and MUT rat DNA sequences confirms the single point mutation c.736 T > G in the rat Elovl4 genome. Box and arrows indicate site of gene editing. a. MUT. b. HET. c. WT. (PNG 1558 kb) [file 12035_2020_2052_Fig13_ESM.png]

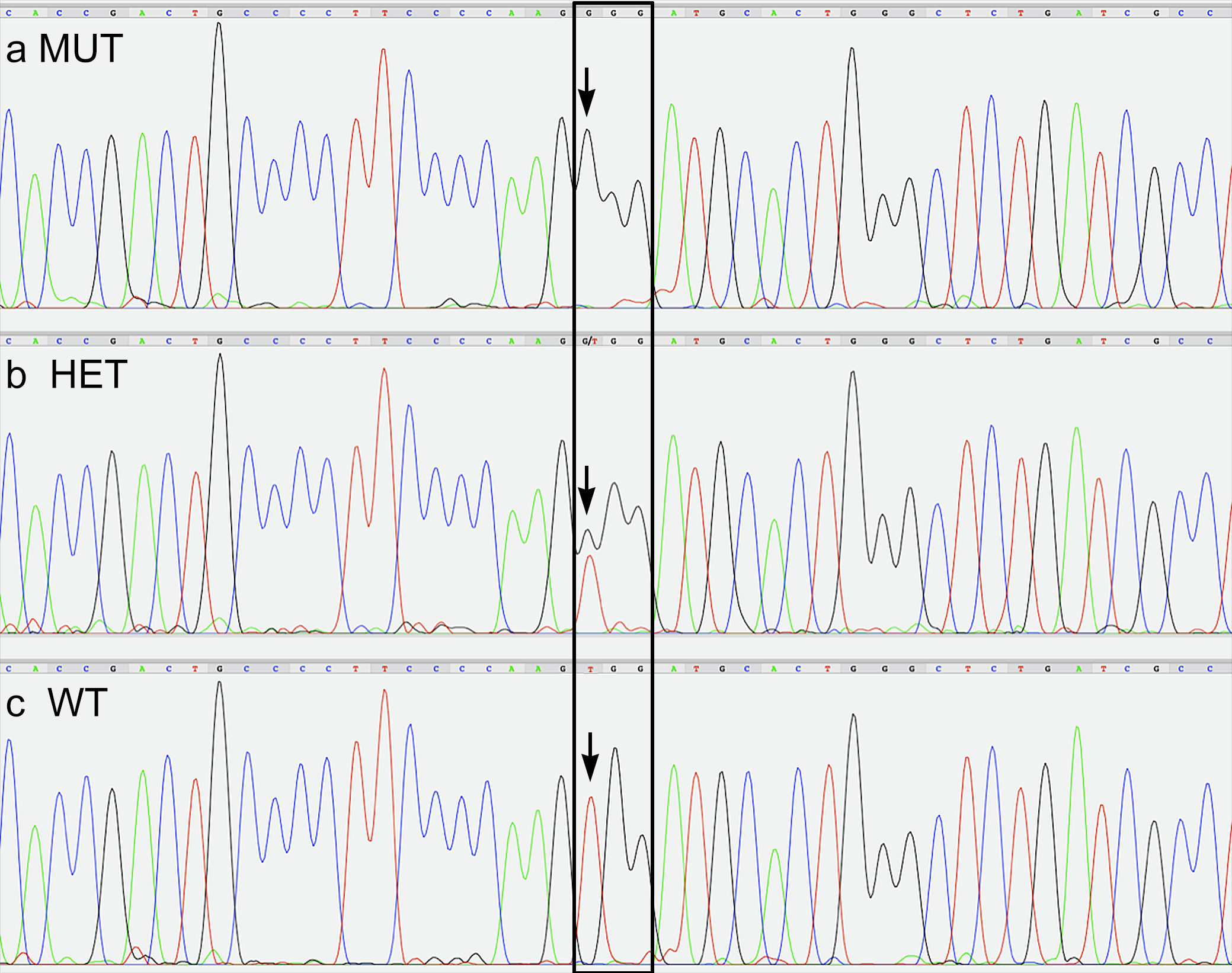

Supplement: Supplementary file 2 — High resolution image (TIF 5700 kb) [file 12035_2020_2052_MOESM1_ESM.tif]

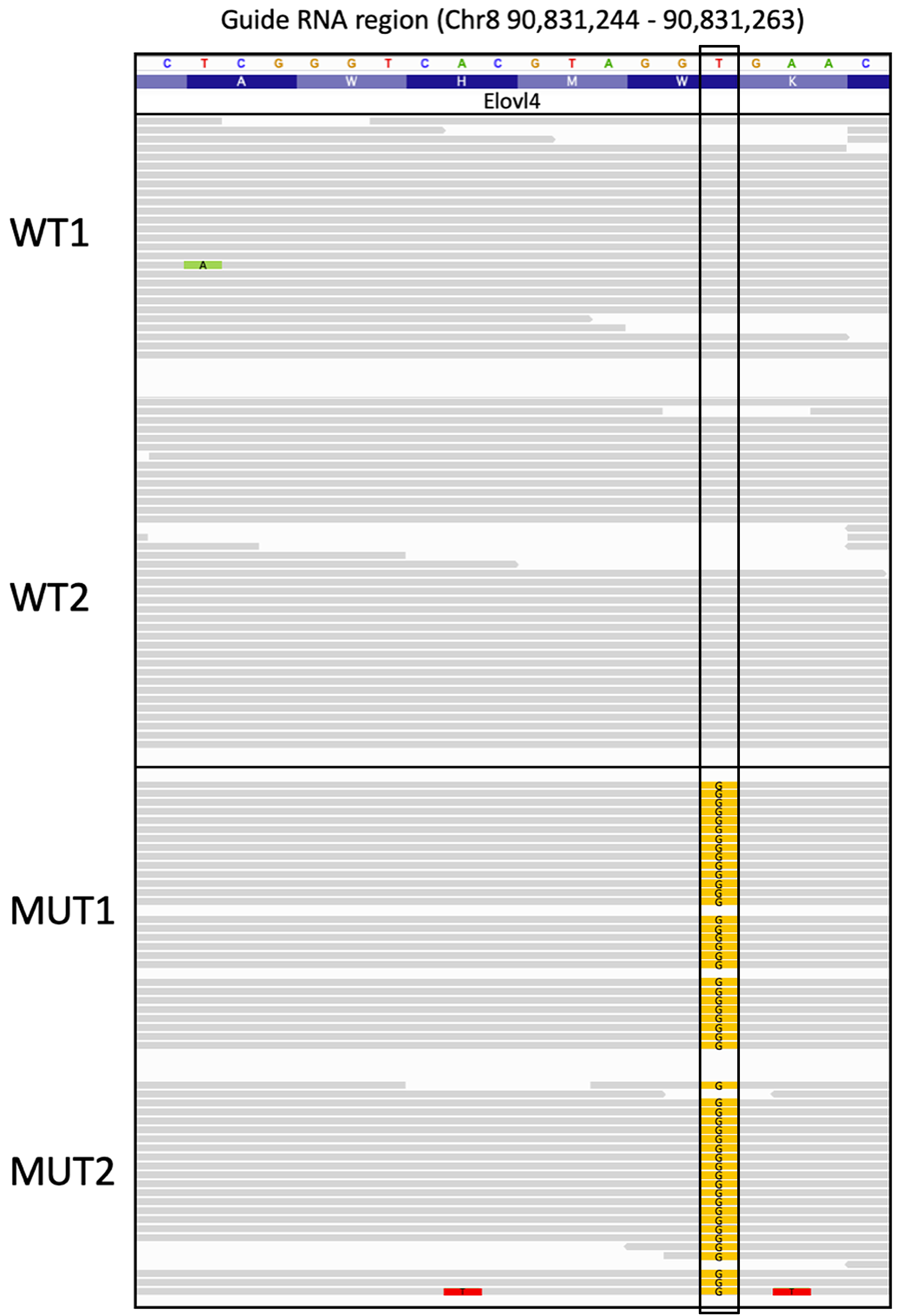

Supplement: Supplementary file 3 — Whole genome sequence analysis. Reading from the 3′-5 primer direction (right to left on the figure), whole genome sequencing of WT and MUT rats confirms knockin of the 736 T > G, p.W246G mutant Elovl4 without any major off target effects in MUT rats (MUT). The box highlights the position of the 736 T > G mutation. Each gray bar represents a NextGen sequence. Colored bases differ from the WT sequence. Bases matching the WT are shown in gray to highlight only mutant bases. Examples of whole genome sequencing from two WT (WT 1 and WT2) and two MUT (MUT1 and MUT2) rats are shown. (PNG 385 kb) [file 12035_2020_2052_Fig14_ESM.png]

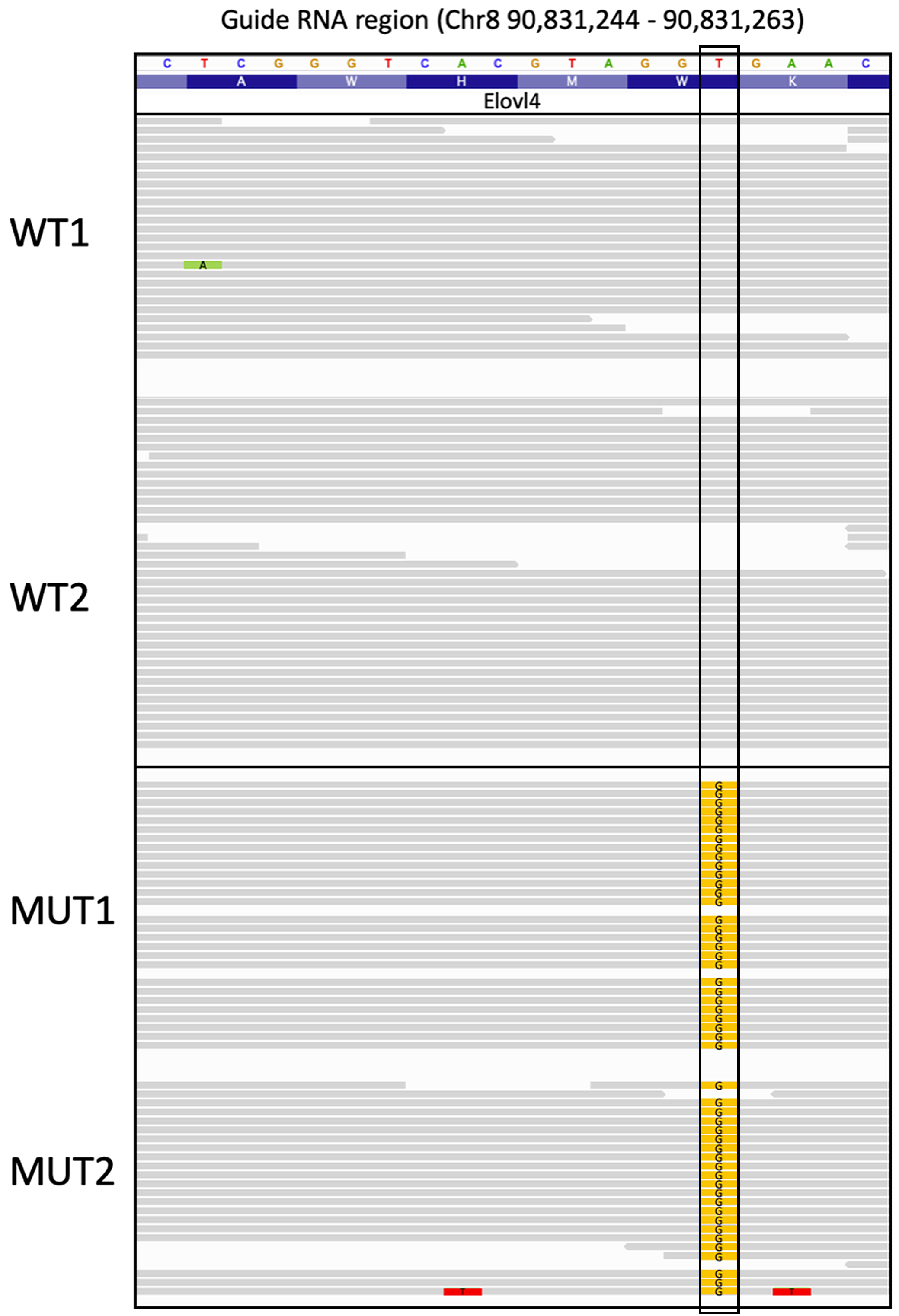

Supplement: Supplementary file 4 — High resolution image (TIF 1852 kb) [file 12035_2020_2052_MOESM2_ESM.tif]

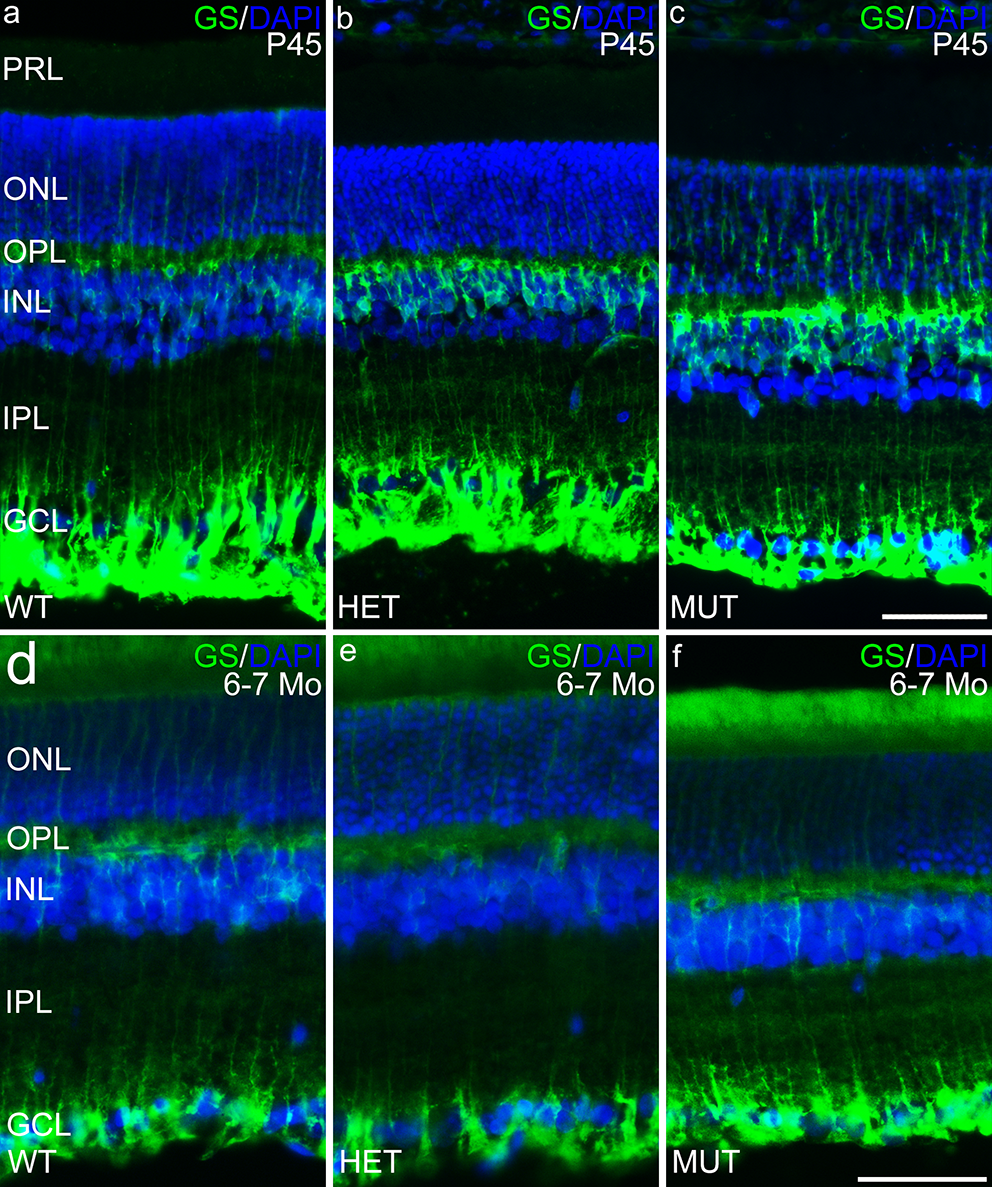

Supplement: Supplementary file 5 — Glutamine synthetase labeling (GS, green) and Müller cell morphology is normal in the WT, HET, and MUT SCA34-KI rat retina. a-c. P45. d-f. 6–7 months of age. Labeling associated with the blood vessels in the retina is non-specific. Retina counterstained with DAPI (blue) to show nuclear layers. PRL, photoreceptor layer; ONL, outer nuclear layer; OPL, outer plexiform layer; INL, inner nuclear layer; IPL, inner plexiform layer; GCL, ganglion cell layer. Scale bars = 50 μm for each row. (PNG 1546 kb) [file 12035_2020_2052_Fig15_ESM.png]

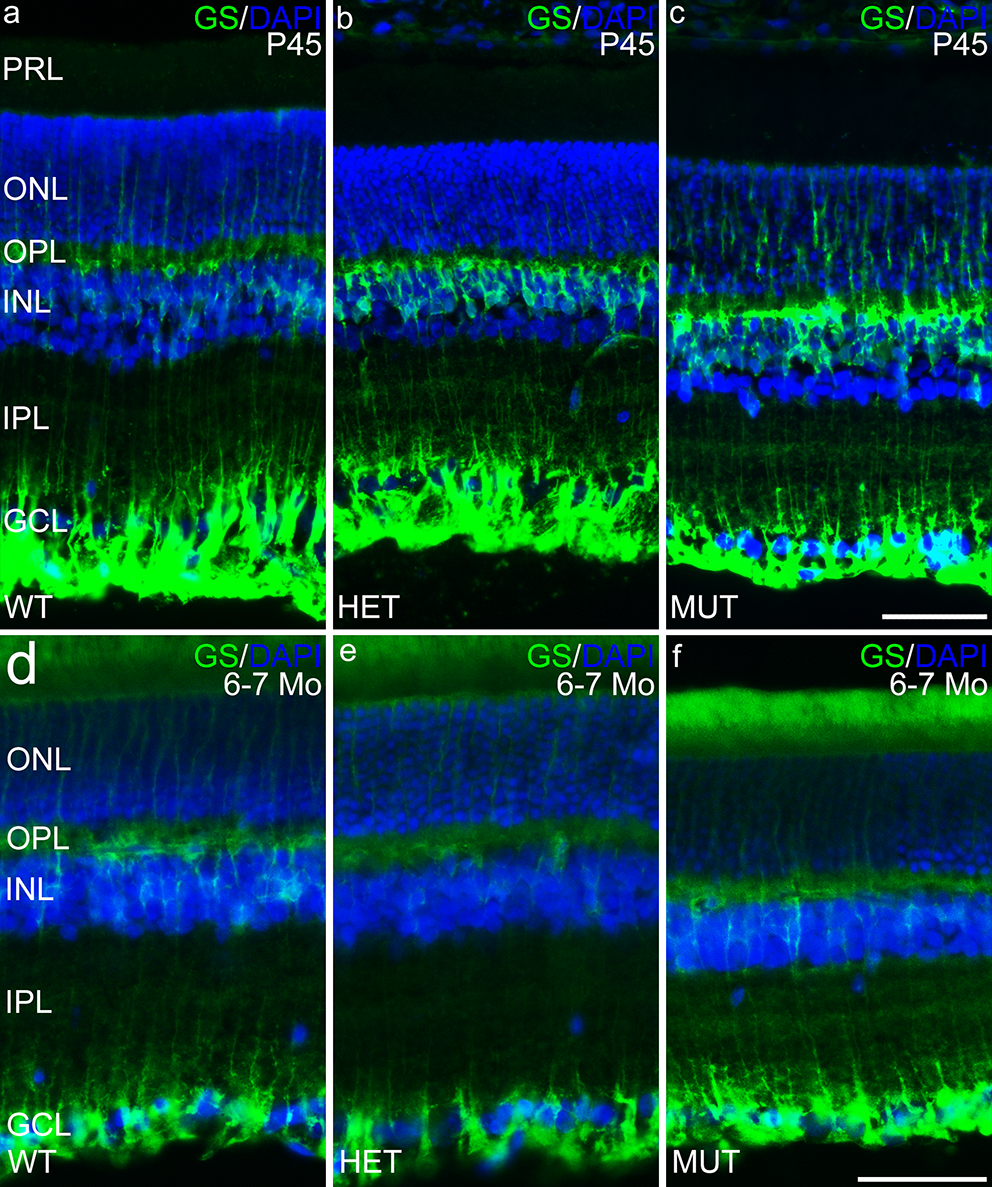

Supplement: Supplementary file 6 — High resolution image (TIF 2002 kb) [file 12035_2020_2052_MOESM3_ESM.tif]

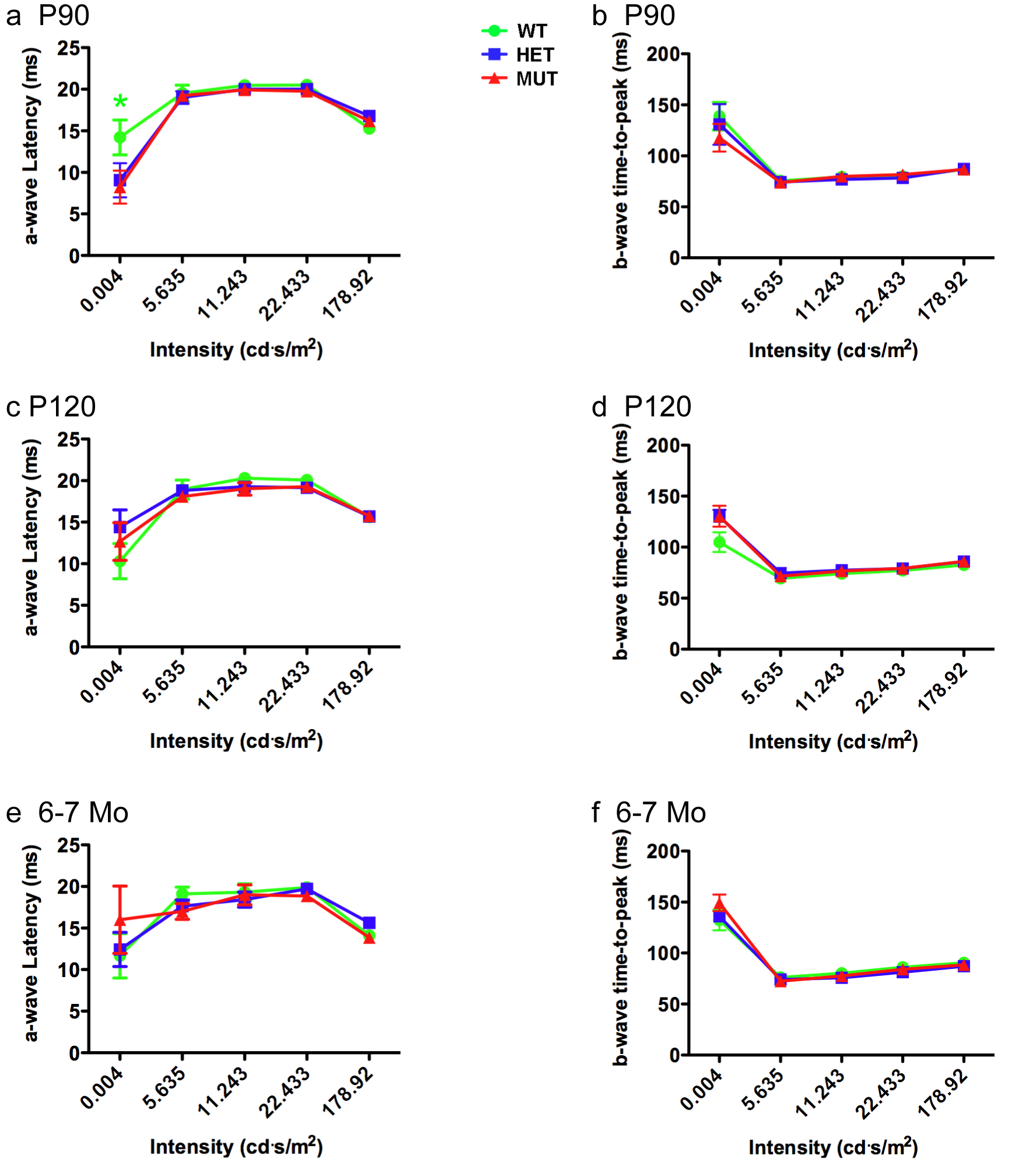

Supplement: Supplementary file 7 — Analysis of scotopic a-wave latency (a,c,e) and b-wave time-to-peak (b,d,f) at P90, P120, and P180 in WT, HET and MUT SCA34-KI rats. (Data shown as mean ± SEM. One-way ANOVA with Tukey’s post-hoc test. Asterisk indicates statistical significance at p < 0.05 for WT response compared to HET and MUT response at P90 in panel A. P90: 14 WT, 14 HET, 17 MUT. P120: 13 WT, 15 HET, 15 MUT. 6–7 Mo: 9 WT, 14 HET, 7 MUT). (PNG 397 kb) [file 12035_2020_2052_Fig16_ESM.png]

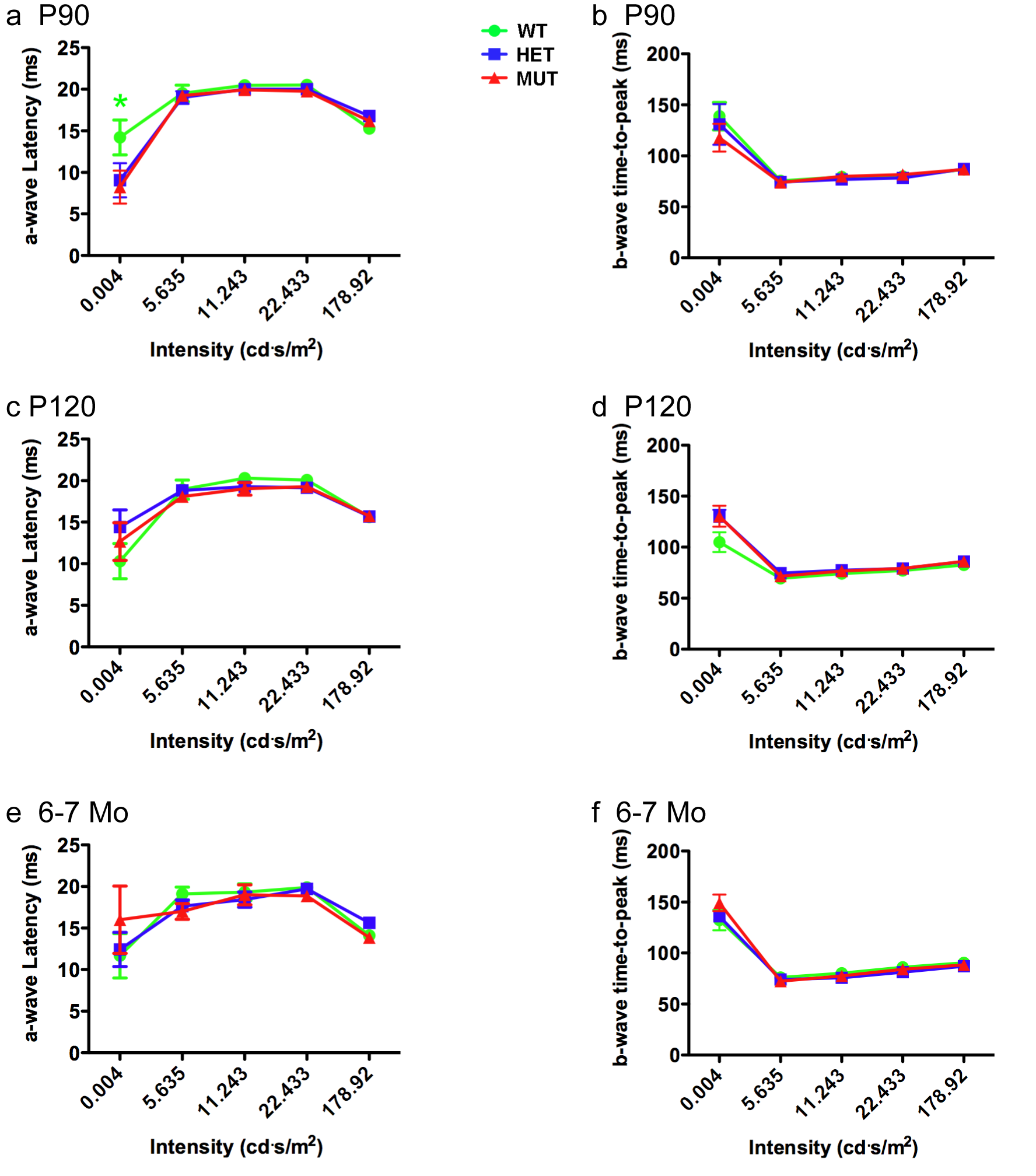

Supplement: Supplementary file 8 — High resolution image (TIF 566 kb) [file 12035_2020_2052_MOESM4_ESM.tif]

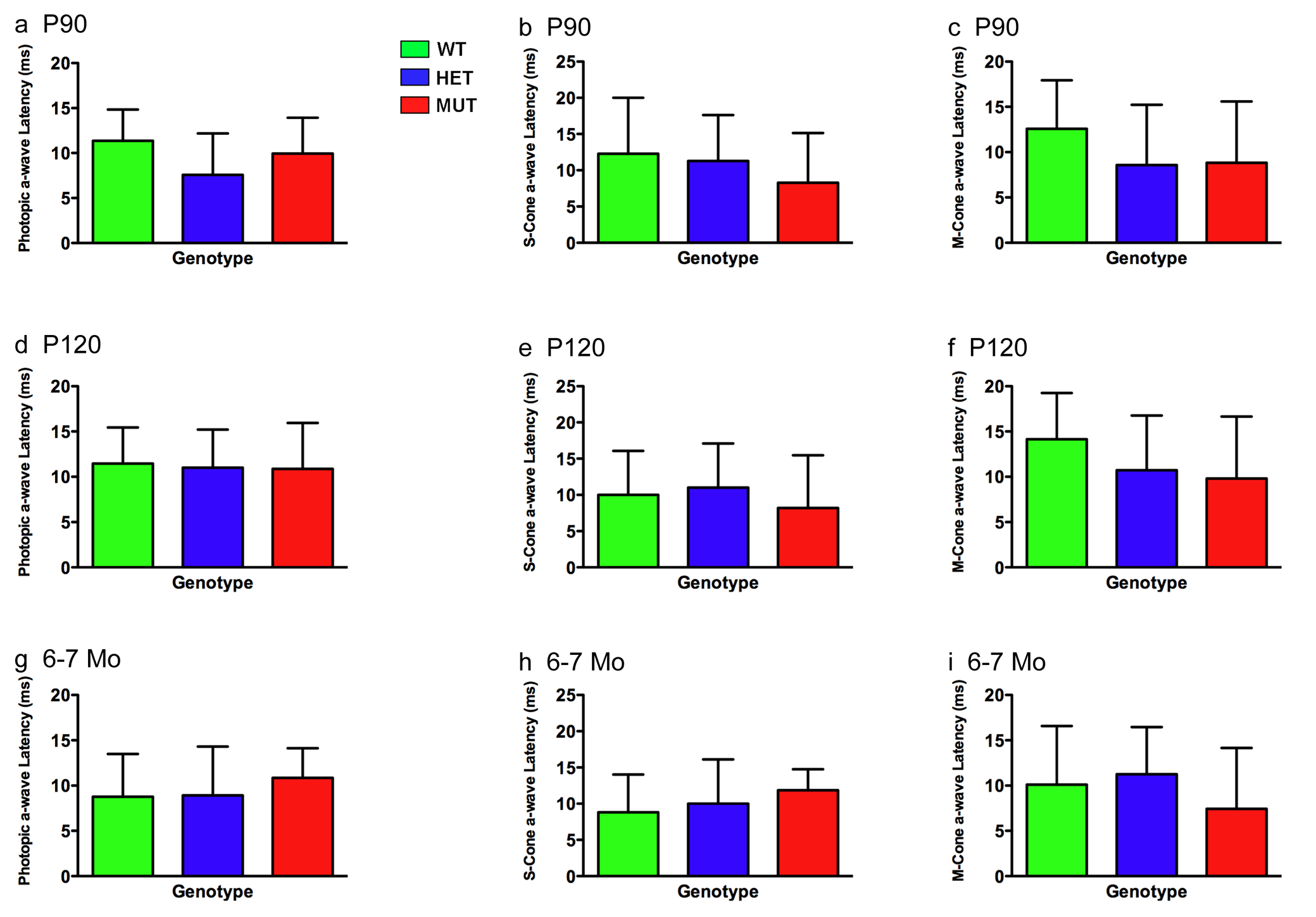

Supplement: Supplementary file 9 — Analysis of photopic (S + M-cone)-driven a-wave latency (a,d,g), S-cone-driven a-wave latency (b,e,h), and M-cone-driven a-wave latency (c,f,i) at P90, P120, and 6–7 Mo in WT, HET, and MUT SCA34-KI rats. (Data shown as mean ± SEM. One-way ANOVA with Tukey’s post-hoc test. P90: 14 WT, 14 HET, 17 MUT. P120: 13 WT, 15 HET, 15 MUT. 6–7 Mo: 9 WT, 14 HET, 7 MUT). (PNG 253 kb) [file 12035_2020_2052_Fig17_ESM.png]

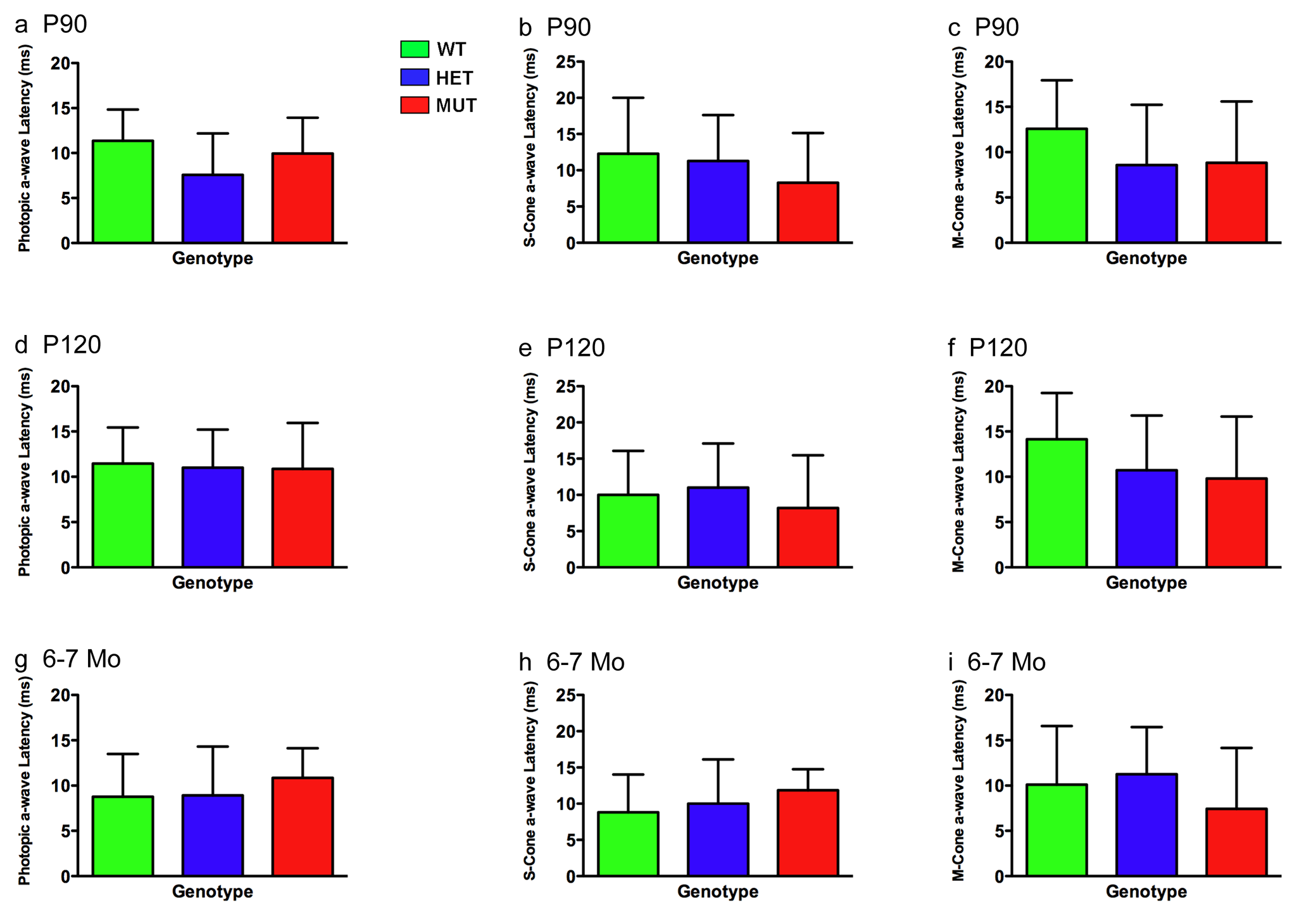

Supplement: Supplementary file 10 — High resolution image (TIF 474 kb) [file 12035_2020_2052_MOESM5_ESM.tif]

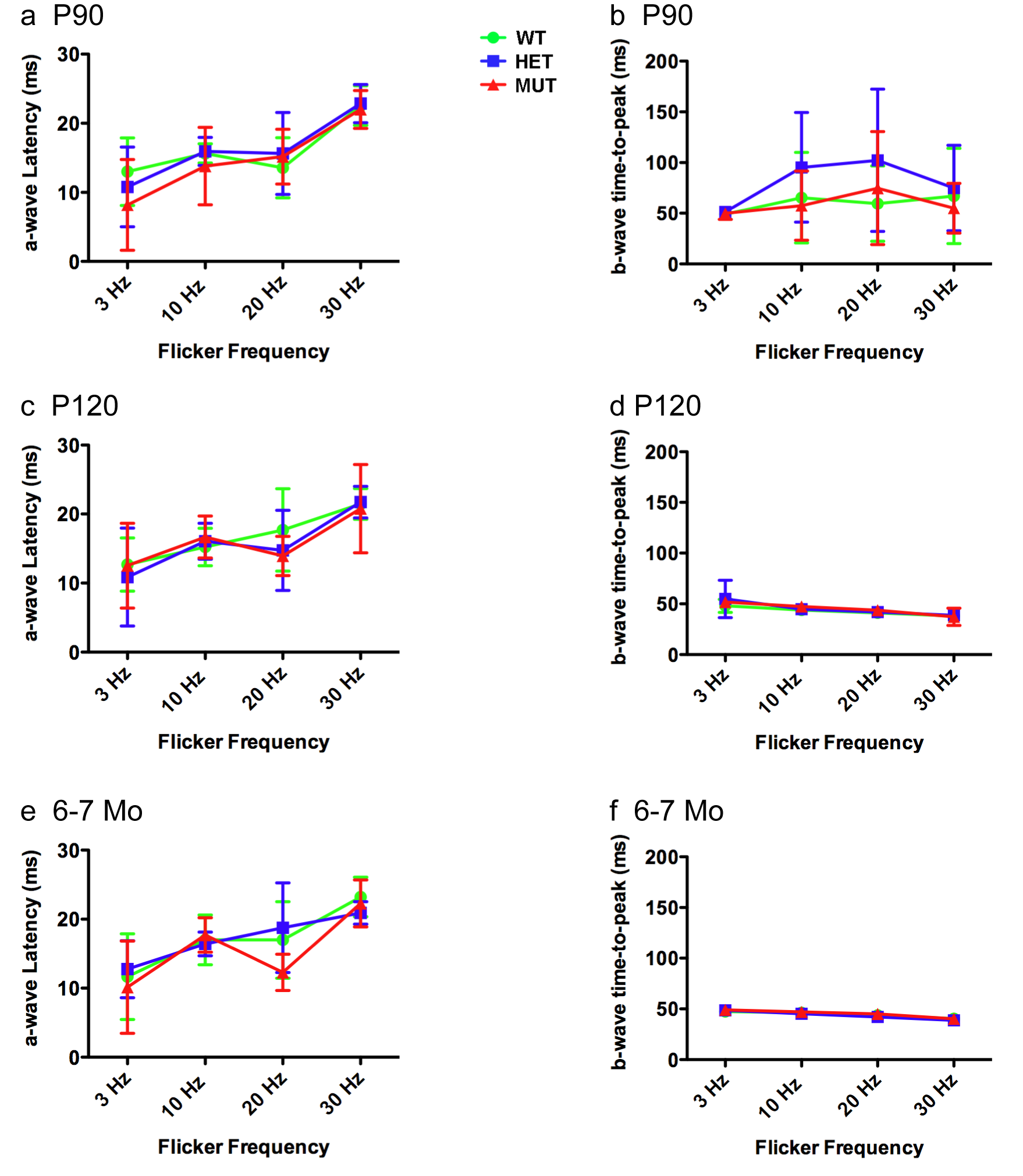

Supplement: Supplementary file 11 — Analysis of flicker ERG a-wave latency (a,c,e), b-wave time to peak (b,d,f) at P90, P120, and 6–7 Mo in WT, HET, and MUT rats. (Data shown as mean ± SEM. One-way ANOVA with Tukey’s post-hoc test. P90: 14 WT, 14 HET, 17 MUT. P120: 13 WT, 15 HET, 15 MUT. 6–7 Mo: 9 WT, 14 HET, 7 MUT). (PNG 334 kb) [file 12035_2020_2052_Fig18_ESM.png]

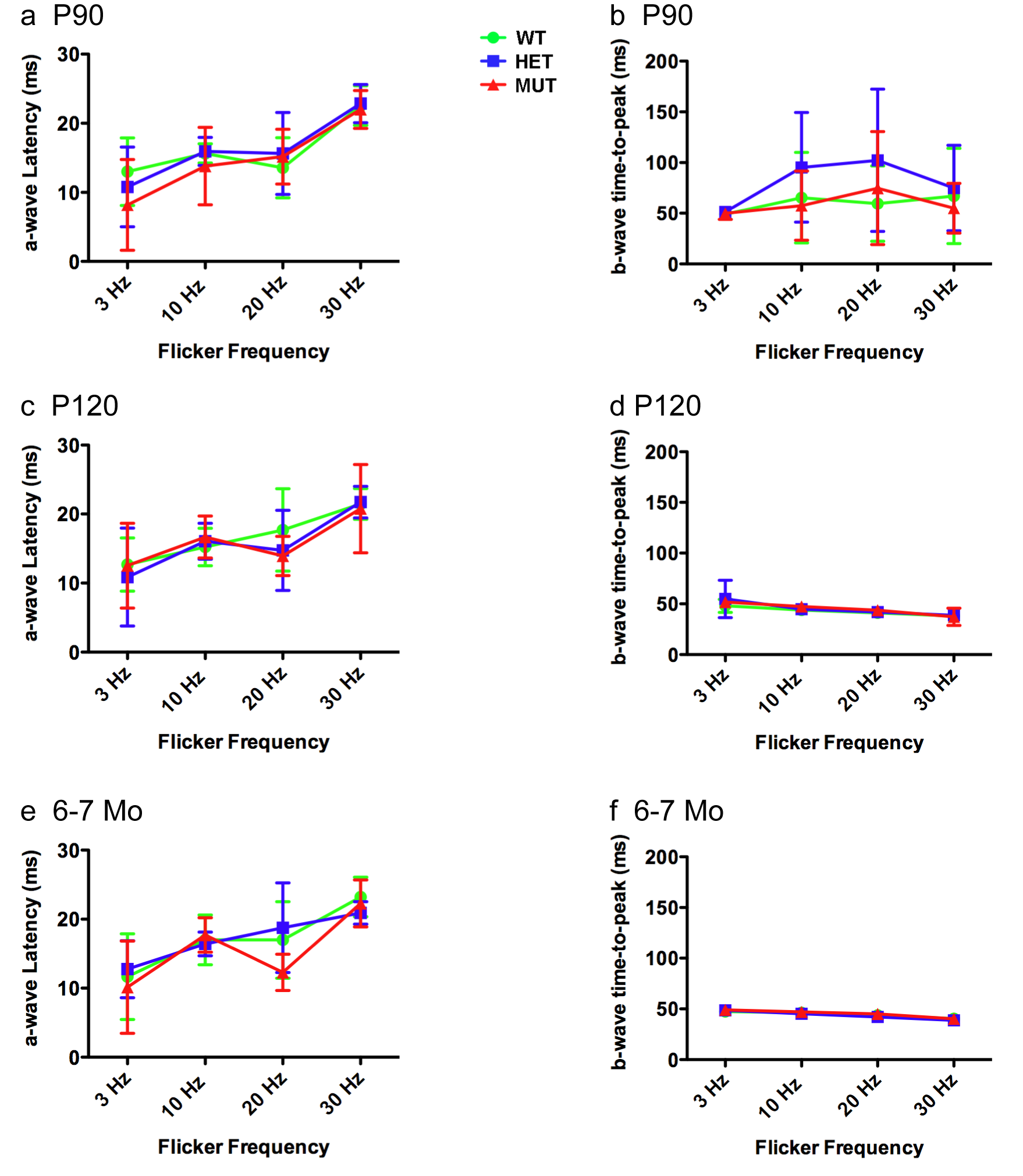

Supplement: Supplementary file 12 — High resolution image (TIF 503 kb) [file 12035_2020_2052_MOESM6_ESM.tif]

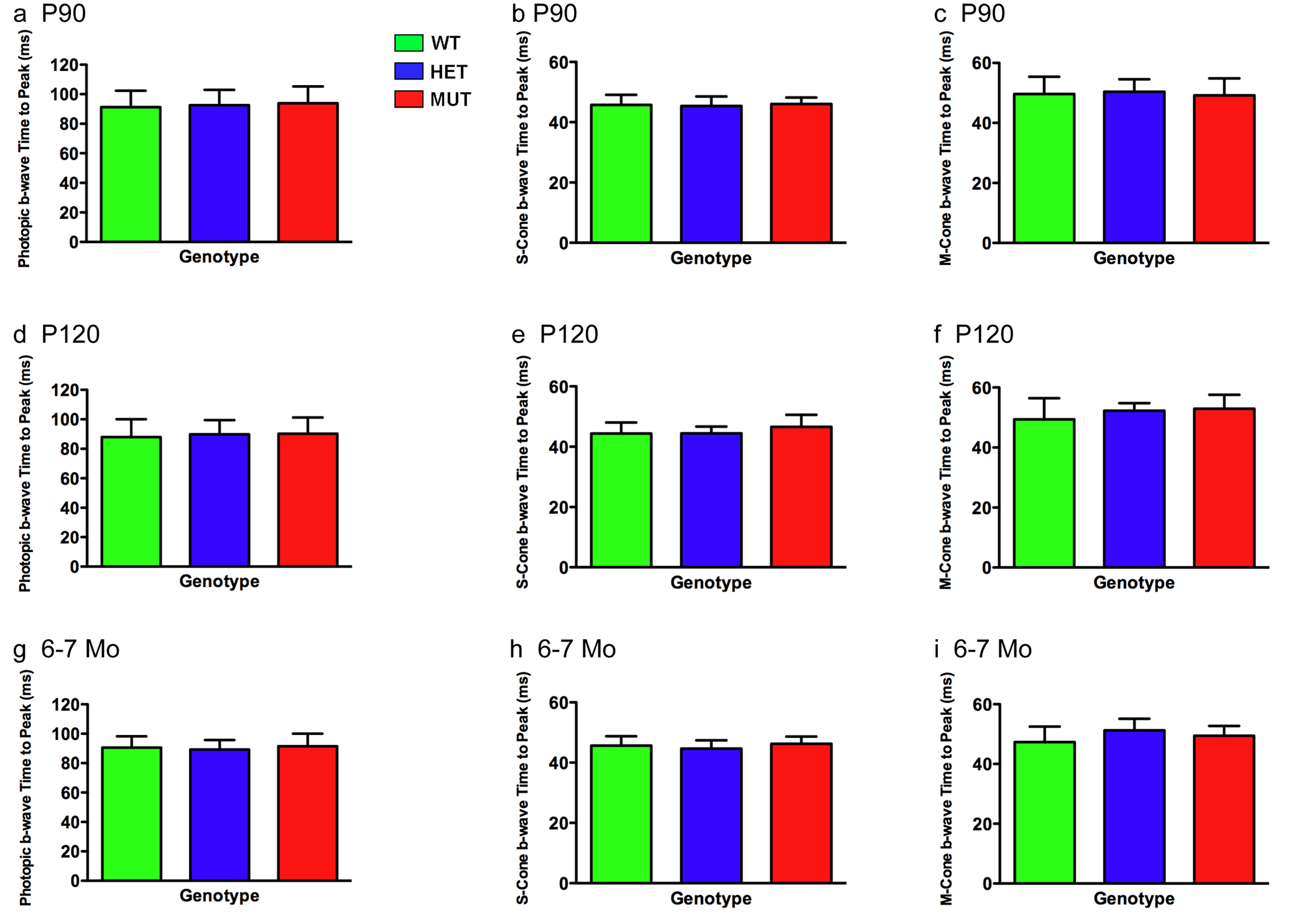

Supplement: Supplementary file 13 — Analysis of photopic (S + M-cone)-driven b-wave time-to-peak (a,d,g), S-cone-driven b-wave time-to-peak (b,e,h), and M-cone-driven b-wave time-to-peak (c,g,i) at P90, P120, and 6–7 Mo in WT, HET, and MUT SCA34-KI rats. (Data shown as mean ± SEM. One-way ANOVA with Tukey’s post-hoc test. P90: 14 WT, 14 HET, 17 MUT. P120: 13 WT, 15 HET, 15 MUT. 6–7 Mo: 9 WT, 14 HET, 7 MUT). (PNG 280 kb) [file 12035_2020_2052_Fig19_ESM.png]

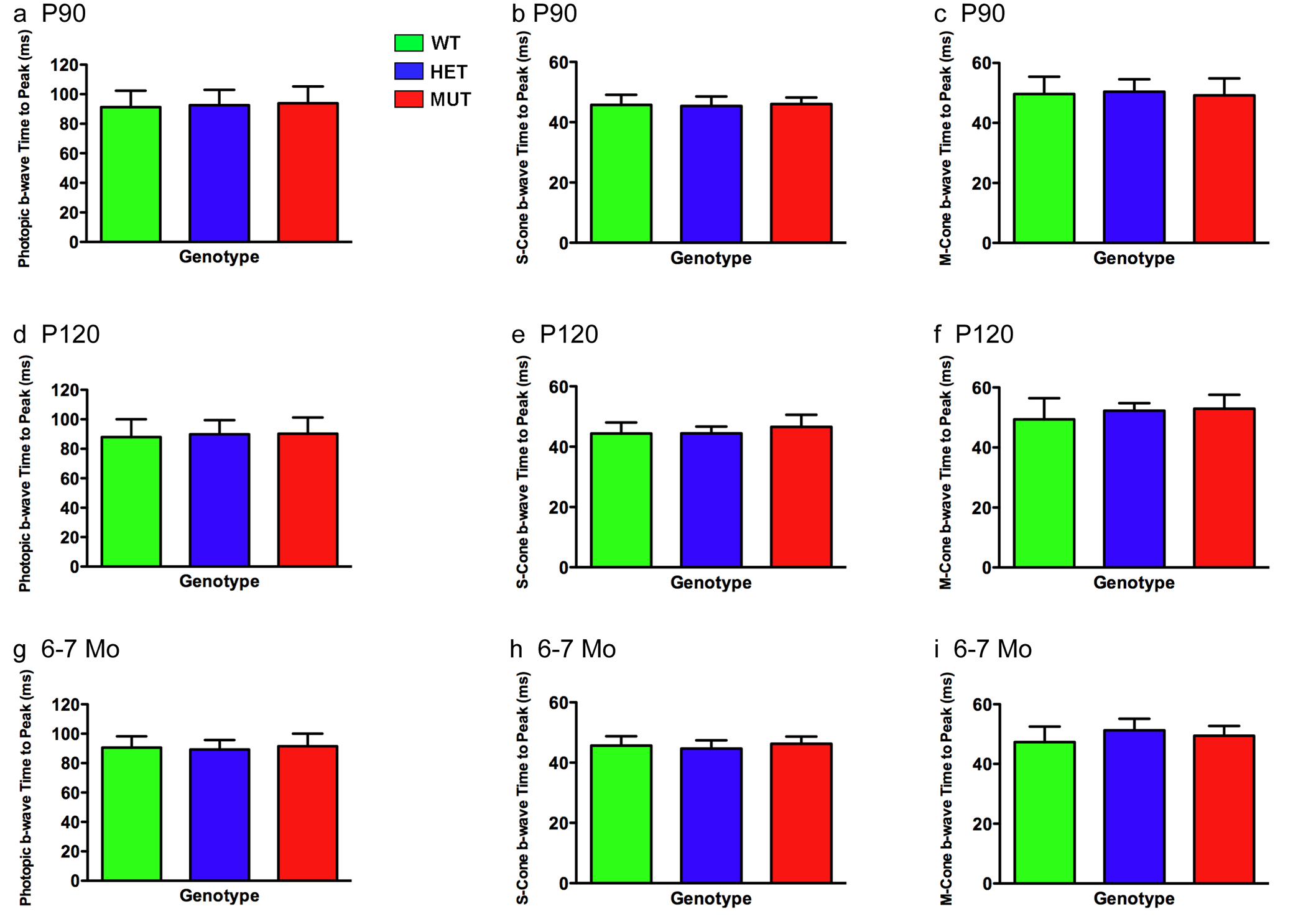

Supplement: Supplementary file 14 — High resolution image (TIF 561 kb) [file 12035_2020_2052_MOESM7_ESM.tif]
