# Supplementary material for: The Elovl4 Spinocerebellar Ataxia-34 Mutation 736T>G (p.W246G) Impairs Retinal Function in the Absence of Photoreceptor Degeneration
Source: Mol Neurobiol. 2020 Aug 11;57(11):4735–53. doi: 10.1007/s12035-020-02052-8 (PMC7515967; doi:10.1007/s12035-020-02052-8)
Supplement: Supplementary file 15 — (PDF 56 kb) [file 12035_2020_2052_MOESM8_ESM.pdf]

Supplemental Table 1. Polyunsaturated fatty acids in retinal phosphatidylcholine (PC)

Phospholipid class	Species	wt/wt	wt/SCA34	SCA34/SCA34
PC	PC 32:00	14.06 +/- 0.72	13.81 +/- 1.23	14.25 +/- 0.78
PC	PC 32:01	2.53 +/- 0.15	2.1 +/- 0.16	2.23 +/- 0.37
PC	PC 34:00	4.87 +/- 0.2	4.7 +/- 0.81	4.86 +/- 0.16
PC	PC 34:01	16.68 +/- 0.59	17.18 +/- 0.75	18.1 +/- 1.05****
PC	PC 34:02	2.23 +/- 0.2	2.04 +/- 0.18	2.08 +/- 0.11
PC	PC 36:01	7.6 +/- 0.44	7.68 +/- 0.38	7.7 +/- 0.07
PC	PC 36:02	2.05 +/- 0.1	2.06 +/- 0.03	2.04 +/- 0.2
PC	PC 36:04	3.63 +/- 0.18	3.42 +/- 0.38	3.79 +/- 0.22
PC	PC 38:04	5.02 +/- 0.1	4.91 +/- 0.17	5.34 +/- 0.38
PC	PC 38:06	7.3 +/- 0.45	7.1 +/- 0.37	6.9 +/- 0.37
PC	PC 40:06	18.5 +/- 0.42	19.12 +/- 0.45	18 +/- 0.54##
PC	PC 40:07	1.78 +/- 0.15	1.63 +/- 0.05	1.45 +/- 0.03
PC	PC 44:12	6.21 +/- 0.15	6.33 +/- 0.44	5.85 +/- 0.58
PC VLC-PUFA	PC 50:10	0.03 +/- 0.01	0.03 +/- 0.01	0.05 +/- 0.01
PC VLC-PUFA	PC 50:11	0.03 +/- 0.03	0.06 +/- 0.02	0.05 +/- 0.01
PC VLC-PUFA	PC 50:12	0.1 +/- 0.01	0.13 +/- 0.03	0.15 +/- 0.01
PC VLC-PUFA	PC 52:10	0.12 +/- 0.05	0.13 +/- 0.03	0.19 +/- 0.02
PC VLC-PUFA	PC 52:11	0.09 +/- 0.02	0.1 +/- 0.04	0.14 +/- 0.02
PC VLC-PUFA	PC 52:12	0.2 +/- 0.08	0.3 +/- 0.03	0.44 +/- 0.04
PC VLC-PUFA	PC 54:10	0.32 +/- 0.02	0.28 +/- 0.01	0.19 +/- 0.02
PC VLC-PUFA	PC 54:11	0.64 +/- 0.07	0.59 +/- 0.13	0.54 +/- 0.07
PC VLC-PUFA	PC 54:12	2.82 +/- 0.63	3.79 +/- 0.28*	4.16 +/- 0.14***
PC VLC-PUFA	PC 56:10	0.12 +/- 0.02	0.09 +/- 0.03	0.04 +/- 0
PC VLC-PUFA	PC 56:11	0.53 +/- 0.18	0.41 +/- 0.13	0.17 +/- 0.03
PC VLC-PUFA	PC 56:12	2.3 +/- 0.46	1.85 +/- 0.13	1.17 +/- 0.25**
PC VLC-PUFA	PC 58:10	0 +/- 0.01	0.01 +/- 0.01	0 +/- 0
PC VLC-PUFA	PC 58:11	0.02 +/- 0	0.02 +/- 0.01	0.01 +/- 0.01
PC VLC-PUFA	PC 58:12	0.22 +/- 0.07	0.14 +/- 0.01	0.09 +/- 0.02
PC VLC-PUFA	Σ PC vlc-pufa	7.54 +/- 0.61	7.92 +/- 0.51	7.4 +/- 0.38

Data shown as mean +/- standard deviation. Statistical analysis by 1-way ANOVA with Tukey's posthoc test.

*, differs from wt/wt at level of $p < 0.05$.
**, differs from wt/wt at level of $p < 0.01$.
***, differs from wt/wt at level of $p < 0.001$.
****, differs from wt/wt at level of $p < 0.0001$.

##, differs from wt/SCA34 at level of $p < 0.01$.
