# Supplementary material for: The Elovl4 Spinocerebellar Ataxia-34 Mutation 736T>G (p.W246G) Impairs Retinal Function in the Absence of Photoreceptor Degeneration
Source: Mol Neurobiol. 2020 Aug 11;57(11):4735–53. doi: 10.1007/s12035-020-02052-8 (PMC7515967; doi:10.1007/s12035-020-02052-8)
Supplement: Supplementary file 16 — (PDF 53 kb) [file 12035_2020_2052_MOESM9_ESM.pdf]

Supplemental Table 2. Polyunsaturated fatty acids in retinal phosphatidylethanolamine (PE)

Phospholipid class	Species	wt/wt	wt/SCA34	SCA34/SCA34
PE	PE 34:01	0.93 +/- 0.06	1.04 +/- 0.13	0.98 +/- 0.08
PE	PE 36:01	1.75 +/- 0.15	1.69 +/- 0.21	1.85 +/- 0.05
PE	PE 36:02	0.64 +/- 0.09	0.69 +/- 0.1	0.76 +/- 0.14
PE	PE 36:04	0.8 +/- 0.03	0.76 +/- 0.06	0.85 +/- 0.11
PE	PE 38:04	6.64 +/- 0.55	6.13 +/- 0.16	6.78 +/- 0.3
PE	PE 38:05	0.69 +/- 0.05	0.94 +/- 0.18	0.79 +/- 0.07
PE	PE 38:06	9.23 +/- 0.18	9.18 +/- 0.45	9.3 +/- 0.62
PE	PE 40:04	1.87 +/- 0.29	1.83 +/- 0.21	2.07 +/- 0.06
PE	PE 40:05	0.93 +/- 0.12	1.03 +/- 0.2	1.33 +/- 0.48
PE	PE 40:05p	0.84 +/- 0.1	0.8 +/- 0.05	0.69 +/- 0.17
PE	PE 40:06	48.61 +/- 1.02	47.47 +/- 1.33*	47.08 +/- 0.38**** ####
PE	PE 40:07	3.45 +/- 0.28	3.3 +/- 0.58	3.45 +/- 0.51
PE	PE 40:08	0.51 +/- 0.05	0.51 +/- 0.17	0.52 +/- 0.12
PE	PE 42:08	0.6 +/- 0.25	0.86 +/- 0.23	1.25 +/- 0.17
PE	PE 42:10	1.35 +/- 0.2	1.35 +/- 0.19	1.12 +/- 0.21
PE	PE 44:10	0.58 +/- 0.15	0.52 +/- 0.07	0.59 +/- 0.18
PE	PE 44:11	0.49 +/- 0.16	0.8 +/- 0.14	0.8 +/- 0.11
PE	PE 44:12	18.71 +/- 0.63	19.22 +/- 0.76	17.63 +/- 1.08*
PE	PE 46:12	0.55 +/- 0.07	0.74 +/- 0.08	0.71 +/- 0.11
PE VLC-PUFA	Σ PE vlc-pufa	none detected	none detected	none detected

Data shown as mean +/- standard deviation. Statistical analysis by 1-way ANOVA with Tukey's posthoc test.

*, differs from wt/wt at level of p < 0.05.
****, differs from wt/wt at level of p < 0.0001.

####, differs from wt/SCA34 at level of p < 0.0001.
