# Supplementary material for: The Elovl4 Spinocerebellar Ataxia-34 Mutation 736T>G (p.W246G) Impairs Retinal Function in the Absence of Photoreceptor Degeneration
Source: Mol Neurobiol. 2020 Aug 11;57(11):4735–53. doi: 10.1007/s12035-020-02052-8 (PMC7515967; doi:10.1007/s12035-020-02052-8)
Supplement: Supplementary file 17 — (PDF 49 kb) [file 12035_2020_2052_MOESM10_ESM.pdf]

Supplemental Table 3. Polyunsaturated fatty acids in retinal phosphatidylserine (PS)

Phospholipid class	Species	wt/wt	wt/SCA34	SCA34/SCA34
PS	PS 38:04	3.58 +/- 0.61	3.19 +/- 0.34	3.33 +/- 0.56
PS	PS 40:04	4.05 +/- 0.16	4.25 +/- 0.87	4.66 +/- 0.87
PS	PS 40:05	2.5 +/- 0.47	2.83 +/- 0.8	2.57 +/- 0.97
PS	PS 40:06	53.91 +/- 0.47	53.37 +/- 4.85	53.56 +/- 3.64
PS	PS 42:09	2.06 +/- 0.65	1.8 +/- 0.65	1.61 +/- 0.57
PS	PS 44:10	3.2 +/- 0.08	3.42 +/- 1.29	3.22 +/- 0.27
PS	PS 44:11	2.31 +/- 0.66	1.56 +/- 0.39	1.97 +/- 0.41
PS	PS 44:12	18.67 +/- 1.05	18.73 +/- 0.93	17.3 +/- 3.06
PS	PS 46:10	2.09 +/- 0.66	2.15 +/- 0.34	1.73 +/- 0.42
PS	PS 46:11	1.88 +/- 0.31	2.28 +/- 0.82	3.21 +/- 0.71
PS VLC-PUFA	Σ PS vlc-pufa	none detected	none detected	none detected

Data shown as mean +/- standard deviation. Statistical analysis by 1-way ANOVA with Tukey's posthoc test. No statistically significant differences detected.
