# Supplementary material for: The Elovl4 Spinocerebellar Ataxia-34 Mutation 736T>G (p.W246G) Impairs Retinal Function in the Absence of Photoreceptor Degeneration
Source: Mol Neurobiol. 2020 Aug 11;57(11):4735–53. doi: 10.1007/s12035-020-02052-8 (PMC7515967; doi:10.1007/s12035-020-02052-8)
Supplement: Supplementary file 18 — (PDF 42 kb) [file 12035_2020_2052_MOESM11_ESM.pdf]

Supplemental Table 4. Levels of key saturated fatty acids in the skin

Species	Class	wt/wt	wt/SCA34	SCA34/SCA34
24:0	Precursor	8.38 +/- 1.26	10.01 +/- 1.17**	10.8 +/- 0.99***
26:0	VLC-SFA precursor	1.73 +/- 0.12	1.84 +/- 0.18	1.97 +/- 0.20
28:0	VLC-SFA	0.17 +/- 0.01	0.16 +/- 0.03	0.09 +/- 0.02*,#
30:0	VLC-SFA	0.12 +/- 0.02	0.09 +/- 0.01	*0.01 +/- 0.00*,#
28:0+30:0	VLC-SFA	0.29 +/- 0.03	0.25 +/- 0.02	*0.1 +/- 0.01*,#

Data shown as mean +/- standard deviation. Statistical analysis by 1-way ANOVA with Tukey's posthoc test.

*, differs from wt/wt at level of $p < 0.05$

**, differs from wt/wt at level of $p < 0.01$

***, differs from wt/wt at level of $p < 0.001$

#, differs from wt/SCA34 at level of $p < 0.05$
